# Detection of Molecular Paths Associated with Insulitis and Type 1 Diabetes in Non-Obese Diabetic Mouse

**DOI:** 10.1371/journal.pone.0007323

**Published:** 2009-10-02

**Authors:** Erno Lindfors, Peddinti V. Gopalacharyulu, Eran Halperin, Matej Orešič

**Affiliations:** 1 VTT Technical Research Centre of Finland, Espoo, Finland; 2 International Computer Science Institute, Berkeley, California, United States of America; University of Miami, United States of America

## Abstract

Recent clinical evidence suggests important role of lipid and amino acid metabolism in early pre-autoimmune stages of type 1 diabetes pathogenesis. We study the molecular paths associated with the incidence of insulitis and type 1 diabetes in the Non-Obese Diabetic (NOD) mouse model using available gene expression data from the pancreatic tissue from young pre-diabetic mice. We apply a graph-theoretic approach by using a modified color coding algorithm to detect optimal molecular paths associated with specific phenotypes in an integrated biological network encompassing heterogeneous interaction data types. In agreement with our recent clinical findings, we identified a path downregulated in early insulitis involving dihydroxyacetone phosphate acyltransferase (DHAPAT), a key regulator of ether phospholipid synthesis. The pathway involving serine/threonine-protein phosphatase (PP2A), an upstream regulator of lipid metabolism and insulin secretion, was found upregulated in early insulitis. Our findings provide further evidence for an important role of lipid metabolism in early stages of type 1 diabetes pathogenesis, as well as suggest that such dysregulation of lipids and related increased oxidative stress can be tracked to beta cells.

## Introduction

Type 1 diabetes (T1D) is an autoimmune disease that results in destruction of insulin-producing beta cells of the pancreas [1]. The early stages of T1D pathogenesis are characterized by insulitis, an inflammation of the islets of Langerhans of the pancreas caused by the lymphocyte infiltration. Although the seroconversion to islet autoantibody positivity has been the first detectable signal for the onset of autoimmunity and progression towards diabetes [2], the initiators of autoimmune response, mechanisms regulating progress toward beta cell failure and factors determining time of presentation of clinical diabetes are poorly understood.

We recently investigated changes in the serum metabolome prospectively in a unique cohort of children at genetic risk for T1D. Intriguingly, we detected multiple changes related to dysregulation of lipid and amino acid metabolism preceding the autoimmunity and overt T1D [3]. In order to better understand the early diabetes pathogenesis, it would have been therefore of great importance to study the molecular mechanisms behind the early metabolic dysregulation as related to the autoimmune response, an area so far neglected in T1D research.

**Figure 1:**
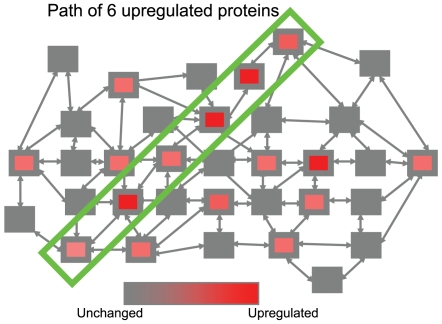
Enriched molecular path detection concept. Illustrative example of path detection in a complex network of interacting entities. An enriched path of 6 entities is highlighted.

Motivated by our clinical findings, here we study molecular paths associated with the incidence of type 1 diabetes (T1D) and insulitis in the Non-Obese Diabetic (NOD) mouse model using the available gene expression data from young pre-diabetic mice [4]. The NOD mouse is a strain whose immune system shares many similarities with human's immune system as well as the autoimmune response [5]. It is therefore widely used in studies aiming to elucidate T1D, although it is also clear that this experimental model may only in part reflect the the immune system and T1D pathogenesis in human [6]. We introduce a method EMPath (*E*nriched *M*olecular *Path* detection) for detection of molecular paths of physical interactions in an integrated network of protein-protein interactions, signal transduction maps and metabolic pathways by applying a modified version of the color coding algorithm [7]. The color coding algorithm was applied previously to detect signaling pathways derived from protein interaction networks [8]. In our approach the phenotype context is achieved by the introduction of path weights based on the network structure combined with the mRNA expression data. Our aim is to detect paths in an integrated network such that up- or down-regulated protein nodes, as estimated by the gene expression data, are significantly over-represented on the path in comparison with the rest of the network (Figure 1).

## Results and Discussion

### Detection of molecular paths associated with insulitis and type 1 diabetes incidence

We applied the EMPath method to an integrated network of protein-protein interactions, signal transduction maps and metabolic pathways where the nodes are proteins or metabolites and the edges are interactions or reactions. In order to study the network in the biological context, we used gene expression information to weight the corresponding protein nodes.

Since our primary aim as related to T1D was to study tissue-specific changes of molecular paths during the early disease pathogenesis, the appropriate study design should include young pre-diabetic mice with selected controls. We searched the T1DBase [9] which hosts T1D related genetic and expression data and identified the study by Vukkadapu *et al.* [4] as the only suitable for our analysis. In addition to that study, there were two other studies available in T1DBase; Chaparro *et al.* [10] and Stanford RoadMap of NOD Type 1 Diabetes (http://fathmanlab.stanford.edu/roadmap_study_design.html). However, we found that Vukkadapu *et al* is more suitable for our analysis than these studies. Chaparro *et al.* contains data from 6-, 9- and 15 week old mice, whereas Vukkadapu *et al.* investigated 3 week old mice. The young mice are more informative for the goals of our study since insulitis is known to occur until 3 or 4 week of age [5]. Standford RoadMap has not yet been published in any journal as of August 2009. However, once available this data will include young mice and will probably provide relevant data in the context of early disease pathogenesis in NOD mice.

In the study by Vukkadapu *et al.* [4], the pancreatic tissue gene expression data is available for four NOD mouse strains from 3 week old animals: BDC2.5/NOD, NOD, BDC2.5/NOD.scid, and NOD.scid. The data analysis in the primary publication was focusing primarily on known T1D-related genes associated with the autoimmune response and inflammation [4]. The four experimental models studied by Vukkadapu *et al.* have differences in the incidence of insulitis and T1D. The BDC2.5/NOD and NOD mice have accelerated and slow insulitis development, respectively. Therefore, comparison of these mouse models may provide information about the pathways associated with early insulitis although as a limitation one should also keep in mind that this not an ideal comparison since genetic factors associated with *e.g.* age and growth are not controlled for. The BDC2.5/NOD.scid model has extremely high diabetes incidence, which develops already at 3–4 weeks of age, whereas the NOD.scid does not develop diabetes. The pathways associated with differences between these two mouse models may thus provide information about mechanisms specific to late insulitis and T1D.

**Figure 2:**
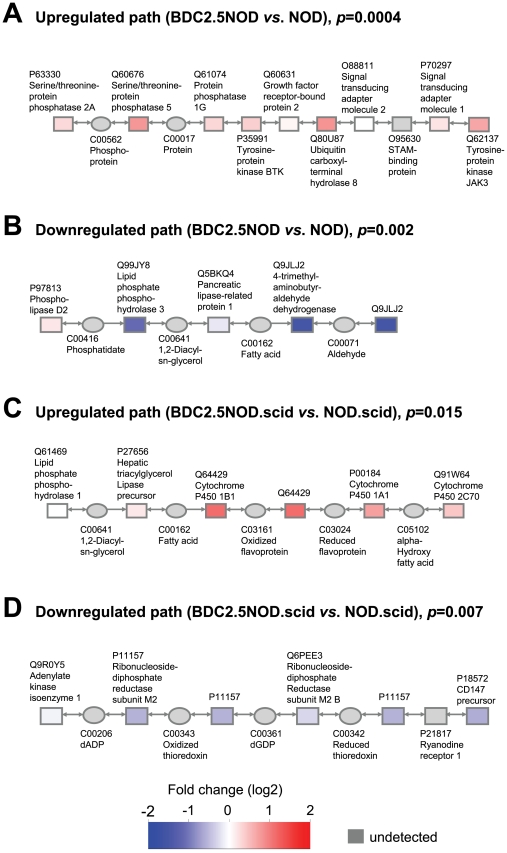
Selected paths significant in different case-control settings. Upregulated (A) and downregulated (B) paths related to insulitis. Upregulated (C) and downregulated (D) paths related to late insulitis and T1D.

We performed path detection for the two comparisons: (1) BDC2.5/NOD *vs*. NOD (early insulitis) and (2) BDC2.5/NOD.vs. NOD.scid (late insulitis and early T1D). We detected multiple optimal paths at *p*<0.025 threshold in both case-control combinations (Figures S2–[Supplementary-material pone.0007323.s012] [Supplementary-material pone.0007323.s013] S5). Selected high scoring paths are shown in Figure 2. Two serine/threonine-protein phosphatases, 2A (PP2A) and 5 (PP5) were members of the most upregulated paths in early insulitis (Figure S2). PP2A and PP5 are known to interact [11], and PP2A is associated with the autoimmune response in systemic lupus erythematosus [12]. Interestingly, PP2A is also a regulator of insulin secretion in pancreatic beta cells [13] and its activation is required for repression of PPARα, a key regulator of genes involved in beta cell fatty acid oxidation [14].

Several paths including lipid metabolism enzymes were found downregulated in early insulitis (Figure S3, Table S1). Lipid phosphate phosphohydrolase 3 (LPP3) hydrolizes specific phospholipids in the lipid membrane, leading to production of *e.g.* diacylglycerols and ceramides [15]. Two of the enzymes of carnitine metabolism, carnitine O-palmitoyltransferase I (CPT1) and 4-trimethyl aminobutyraldehyde dehydrogenase (TMABADH), were also downregulated in the BDC2.5/NOD mice. Interestingly, the dihydroxyacetone phosphate acyltransferase (DHAPAT; Uniprot ID P98192), a key regulator of ether phospholipid synthesis [16], was found in a downregulated path in close proximity of CPT1 (Figure S3).

Two interacting members of the cytochrome P450 family, CYP1B1 and CYP1A1, were found upregulated and present in multiple paths associated with late insulitis and T1D (Figure S4), while basigin (CD147 antigen, also named extracellular matrix metalloproteinase inducer) was found in several downregulated paths (Figure S5). CD147 is a receptor of cyclophilins and is an important messenger of intercellular communication involved also in recruitment of leukocytes from the periphery into tissues during inflammatory responses [17].

As a potential limitation of our approach, in the path detection method presented here we assign weights to nodes based on mRNA expression data and not on protein concentration or direct interaction data. The protein-level data would be ideal for our approach, but such data is generally not available at the global scale such as in transcriptomics studies. We thus use the protein encoding mRNA expression as an approximation, although it is well known that mRNA and corresponding protein level do not always correlate [18]. Although approximate, we believe that use of mRNA expression when protein-level data is unavailable or too sparse is justified and can still provide useful hints about the molecular paths associated with the investigated phenotypes.

### Functional characterization of molecular paths

To better understand the paths detected by EMPath in the context of known pathways, we assessed the functional enrichment of detected paths similarly as previously described [8]. We cross-classified the proteins from a molecular path according to whether or not their encoding genes belong to gene sets obtained from the Molecular Signature Database (MSigDB) [19] and tested if the number of those genes associated with the path is larger than expected by chance using the hypergeometric test. We corrected the *p*-values for multiple comparisons using the False Discovery Rate (FDR) *q-*values. By setting the statistical significance level at FDR *q*<0.05, we identified multiple gene sets over-represented among the detected molecular paths (Table S2). As a summary, the top ten enriched pathways in each of the case-control settings are shown in Table 1.

**Table 1:** Top enriched pathways in insulitis and type 1 diabetes as derived from detected paths.

Gene set	Source	n(P & G)	n(G)	Nominal*p*-value	FDR *q*-value
**Enriched in upregulated paths (BDC2.5/NOD ** ***vs.*** ** NOD)**					
PTDINSPATHWAY	BioCarta	3	19	0.000004	0.000103
HSA00051_FRUCTOSE_AND_MANNOSE_METABOLISM	KEGG	3	27	0.000012	0.000155
HSA00530_AMINOSUGARS_METABOLISM	KEGG	2	16	0.000025	0.000280
GALACTOSE_METABOLISM	GenMAPP	2	20	0.000596	0.003099
HSA00052_GALACTOSE_METABOLISM	KEGG	2	24	0.000863	0.003738
GLUCONEOGENESIS	GenMAPP	2	39	0.002286	0.006604
GLYCOLYSIS	GenMAPP	2	39	0.002286	0.006604
HSA04630_JAK_STAT_SIGNALING_PATHWAY	KEGG	4	100	0.000118	0.008023
HSA04664_FC_EPSILON_RI_SIGNALING_PATHWAY	KEGG	3	62	0.000150	0.008426
GHPATHWAY	BioCarta	2	24	0.000863	0.016104
**Enriched in downregulated paths (BDC2.5/NOD ** ***vs.*** ** NOD)**					
GLYCEROLIPID_METABOLISM	GenMAPP	3	24	<10^−6^	0.000011
STATIN_PATHWAY_PHARMGKB	GenMAPP	2	16	0.000152	0.000557
HSA00565_ETHER_LIPID_METABOLISM	KEGG	2	21	0.000441	0.002093
HSA00071_FATTY_ACID_METABOLISM	KEGG	2	29	0.000847	0.002311
HSA00120_BILE_ACID_BIOSYNTHESIS	KEGG	2	20	0.000399	0.002311
HSA00220_UREA_CYCLE_AND_METABOLISM_OF_AMINO_GROUPS	KEGG	2	21	0.000441	0.002311
HSA00310_LYSINE_DEGRADATION	KEGG	2	29	0.000847	0.002311
HSA00340_HISTIDINE_METABOLISM	KEGG	2	19	0.000359	0.002311
HSA00410_BETA_ALANINE_METABOLISM	KEGG	2	17	0.000286	0.002311
HSA00620_PYRUVATE_METABOLISM	KEGG	2	28	0.000789	0.002311
**Enriched in upregulated paths (BDC2.5/NOD.scid ** ***vs.*** ** NOD.scid)**					
EGFPATHWAY	BioCarta	4	25	<10^−6^	0.000040
HSA04630_JAK_STAT_SIGNALING_PATHWAY	KEGG	5	100	0.000002	0.000102
HSA05213_ENDOMETRIAL_CANCER	KEGG	4	42	0.000004	0.000128
HSA05223_NON_SMALL_CELL_LUNG_CANCER	KEGG	4	43	0.000004	0.000128
CTLA4PATHWAY	BioCarta	3	15	0.000008	0.000131
ERK5PATHWAY	BioCarta	3	16	0.000010	0.000131
HSA05214_GLIOMA	KEGG	4	50	0.000007	0.000131
PTENPATHWAY	BioCarta	3	16	0.000010	0.000131
NGFPATHWAY	BioCarta	3	17	0.000012	0.000140
IGF1PATHWAY	BioCarta	3	18	0.000014	0.000149
**Enriched in downregulated paths (BDC2.5/NOD.scid ** ***vs.*** ** NOD.scid)**					
PYRIMIDINE_METABOLISM	GenMAPP	3	43	0.000010	0.000061
HSA00230_PURINE_METABOLISM	KEGG	3	90	0.000096	0.000334
NDKDYNAMINPATHWAY	BioCarta	2	16	0.000898	0.006367
HSA05110_CHOLERA_INFECTION	KEGG	1	31	0.039815	0.046451

Top ten enriched gene sets at FDR *q*<0.05 defined in the Molecular Signature Database [19], using the gene lists derived from the detected paths (Figures S2–[Supplementary-material pone.0007323.s012] [Supplementary-material pone.0007323.s013] S5). The *p*-value is obtained from the hypergeometric test. Column legend: n(P&G), number of common genes in the detected path and the gene set; n(G), number of genes in the gene set.

It is evident from Table 1 that early insulitis (*i.e.* BDC2.5/NOD strain, as compared to NOD) is associated with altered cell signaling since multiple (de)phosphorilation pathways are affected. In contrast, the lipid metabolism is diminished. The paths associated with late insulitis and T1D in BDC2.5/NOD.scid strain are related to cell communication and related processes, while the nucleotide and nucleoside metabolism, *i.e.*, likely related to cell cycle and DNA repair, is impaired.

### Comparison of path detection with pathway analysis

We performed Gene Set Enrichment Analysis (GSEA) [19] for both case-control comparisons. Table 2 contains the top scored pathways for each strain at FDR *q*<0.05, while a full list of affected pathways at recommended *q*<0.25 is shown in Tables S3–[Supplementary-material pone.0007323.s006] [Supplementary-material pone.0007323.s007] S6. In agreement with earlier analyses [4], both EMPath (Table 1) and GSEA analyses confirmed multiple inflammatory and T cell activation pathways in pancreatic tissue in late insulitis and early T1D. The cell proliferation, division, as well as nucleotide synthesis pathways were found diminished, confirming increasing cell death and DNA damage at this late stage of disease pathogenesis.

**Table 2:** Top scored pathways in GSEA.

Gene set	Size	Enrichment Score	Nominal p-value	FDR q-value	Source
**Downregulated paths (BDC2.5/NOD vs. NOD)**					
HSA03010_RIBOSOME	44	−0.61	0.000466	0.0027	KEGG
WNTPATHWAY	22	−0.63	0.002375	0.0252	BioCarta
HSA00071_FATTY_ACID_METABOLISM	29	−0.58	0.001845	0.0291	KEGG
CALCINEURINPATHWAY	17	−0.64	0.007370	0.0392	BioCarta
PROTEASOMEPATHWAY	21	−0.61	0.004710	0.0418	BioCarta
BILE_ACID_BIOSYNTHESIS	15	−0.65	0.007466	0.0425	GenMAPP
**Upregulated paths (BDC2.5/NOD.scid vs. NOD.scid)**					
HSA04610_COMPLEMENT_AND_COAGULATION_CASCADES	52	0.62	<10–5	0.0022	KEGG
HSA04612_ANTIGEN_PROCESSING_AND_PRESENTATION	33	0.66	<10–5	0.0038	KEGG
HSA04620_TOLL_LIKE_RECEPTOR_SIGNALING_PATHWAY	74	0.54	<10–5	0.0107	KEGG
HSA04060_CYTOKINE_CYTOKINE_RECEPTOR_INTERACTION	169	0.47	<10–5	0.0183	KEGG
NKCELLSPATHWAY	15	0.71	0.002838	0.0310	BioCarta
HSA04940_TYPE_I_DIABETES_MELLITUS	20	0.66	0.002753	0.0353	KEGG
**Downregulated paths (BDC2.5/NOD.scid vs. NOD.scid)**					
CELL_CYCLE_KEGG	58	−0.57	<10–5	0.0031	GenMAPP
CELL_CYCLE	53	−0.56	<10–5	0.0073	GO
UBIQUITIN_MEDIATED_PROTEOLYSIS	20	−0.67	0.000937	0.0096	GenMAPP
G1_TO_S_CELL_CYCLE_REACTOME	54	−0.53	<10–5	0.0112	GenMAPP
HSA00190_OXIDATIVE_PHOSPHORYLATION	86	−0.49	<10–5	0.0113	KEGG
P53PATHWAY	16	−0.70	0.001388	0.0144	BioCarta
PROTEASOMEPATHWAY	21	−0.64	<10–5	0.0174	BioCarta
HSA04120_UBIQUITIN_MEDIATED_PROTEOLYSIS	25	−0.62	0.000473	0.0177	KEGG
HSA04110_CELL_CYCLE	82	−0.47	0.000553	0.0211	KEGG
CARM_ERPATHWAY	19	−0.63	0.004144	0.0279	BioCarta
MRNA_PROCESSING_REACTOME	83	−0.46	<10–5	0.0312	GenMAPP
HSA00510_N_GLYCAN_BIOSYNTHESIS	24	−0.59	0.003738	0.0356	KEGG
G2PATHWAY	18	−0.62	0.004585	0.0475	BioCarta

This table contains top scored gene sets in GSEA for each strain (FDR *q*<0.05). The gene sets studies are the same as in the analysis for Table 1. None of the pathways were significantly upregulated in the BDC2.5/NOD *vs*. NOD comparison using the FDR *q*<0.05 threshold.

In accordance with path detection results, lipid metabolism related pathways (fatty acid metabolism and bile acid synthesis) are downregulated in insulitis, while the cell cycle related pathways are downregulated in T1D (Table 2). The CPT1 and TMABADH found in downregulated paths associated with early insulitis (Table S1) were both among the leading edge genes in the fatty acid metabolism gene set, while TMABADH was also the leading edge in the bile acid synthesis module.

### Meta analysis of findings using T1DBase

To investigate how genes detected by EmPath change in gene expression analyses seen in several other studies, we used the Meta Analysis tool of the T1DBase (http://www.t1dbase.org/page/MetaHome) [9]. As a result, we selected the genes found in the significant molecular paths (Figure 2) and visualized their differential expression across multiple studies available in T1Dbase (Figures S6–[Supplementary-material pone.0007323.s016] [Supplementary-material pone.0007323.s017] S9).

We can see some interesting observations regarding the genes that were involved in our detected paths. DHAPAT (often abbreviated as GNPAT), a gene that was found in paths downregulated in early insulitis in paths detected by EmPath, was also down-regulated in mice deficient for transcriptional regulators FoxA2 and Sox4 [20], [21]. PP2 (also abbreviated as PPP2CA), a gene that was upregulated in early insulitis and type 1 diabetes in paths detected by EmPath, was also upregulated in FoxA2 deficient mouse [20]. Another interesting observation is that the up-/down-regulation of molecular paths in early insulitis in our study matches particularly well with the data from the FoxA2 deficient mouse (Figures S6–S7 and reference [20]). FoxA2 is a transcription factor involved in the regulation of insulin sensitivity [22].

### Ether lipids and oxidative stress in beta cells

**Figure 3:**
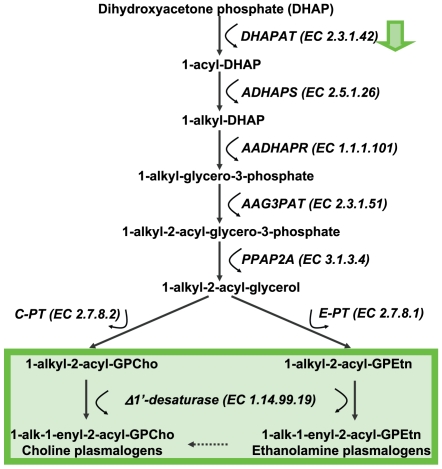
Schematic representation of the steps involved in the biosynthesis of ether phospholipids, including plasmalogens. The lipids found consistently downregulated in serum of children who later developed type 1 diabetes [3] are shown in green box. DHAPAT enzyme is found in the downregulated paths in early insulitis in the present study (green arrow). The first three reactions in the pathway take place in peroxisomes, while the others are catalyzed by microsomal enzyme systems. Other routes for the formation of ether phospholipids may exist [16].

As a most surprising finding from our study, multiple lipid pathways were downregulated in early insulitis (BDC2.5/NOD *vs.* NOD comparison), including the ether lipid metabolism (Table 1). Ether phospholipid synthesis, including synthesis of plasmalogens, starts in peroxisomes and involves esterification of dihydroxyacetone phosphate (DHAP) with a long-chain acyl-CoA ester [16], [23] (Figure 3). This first reaction is catalyzed by dihydroxyacetone phosphate acyltransferase (DHAPAT, EC 2.3.1.42). This reaction appears to be affected in early insulitis, since the path involving DHAPAT is diminished (Tables 1 and S1, Figure S3). The plasmalogens are the most abundant ether phospholipids and may protect cellular functions from oxidative damage [24], [25]. The ether lipids were also found consistently diminished in serum of children who later progressed to type 1 diabetes [3]. Diminished protection against the reactive oxygen species is relevant for T1D since pancreatic beta cells are particularly susceptible to oxidative damage [26], [27]. Further supporting the role of lipids in early insulitis, the enzymes of carnitine metabolism and fatty acid transport to mitochondria (CPT1 and TMABADH) were found in downregulated paths as well.

Previous genetic studies have shown that defective plasmalogen synthesis associates with impaired membrane trafficking [28] although the implications for type 1 diabetes remain to be established [29]. Plasmalogen synthesis-related genes such as DHAPAT clearly need to be evaluated as potential type 1 diabetes susceptibility genes. The complete depletion of ether lipids via a genetic DHAPAT knock-out model leads to a severe phenotype, including arrest of spermatogenesis, development of cataract and defects in central nervous system myelination [30]. In order to study the physiological consequences of altered ether lipid levels as observed in pre-diabetes, one would therefore need to establish experimental models with partial depletion of ether lipids.

### Conclusions

We demonstrated that graph-theoretic approaches such as EMPath are a useful tool for detecting pathways of physical interactions associated with specific disease phenotypes. Our findings from the study of paths associated with early insulitis and T1D are consistent with recent findings from a large scale clinical metabolomics study, suggesting an important role of lipid metabolism in the early stages of T1D pathogenesis. We provide evidence that such dysregulation of lipid metabolism and related oxidative stress may be tracked to beta cells and may thus explain the beta cell loss due to increased oxidative stress. The genes identified as important in early insulitis such as DHAPAT or PP2A clearly need to be investigated further in the context of early T1D pathogenesis as well as for their therapeutic potential.

## Materials and Methods

### Construction of integrated network

We constructed an integrated interaction network by combining protein-protein interactions, signal transduction maps and metabolic pathways in mouse as described previously [31], [32]. The integrated network nodes stand for proteins or metabolites, and edges stand for interactions between nodes. We retrieved protein-protein interactions from BIND [33], MINT [34] and DIP [35], signal transduction interactions from TransPath [36] and biochemical reactions from KEGG [37]. We excluded highly connected cofactors from the network since they do not participate in the actual metabolic conversions as substrates or products. Therefore, their inclusion would connect many metabolically distant enzymes. The excluded cofactors are listed in the Supplementary Table S7.

### Gene expression data

We obtained normalized gene expression data from the T1D dataset [4] from NCBI Gene Expression Omnibus (GEO) database [38] series accession number: GSE1623. We used the samples GSM27446 (BDC2.5/NOD1), GSM27451 (BDC2.5/NOD.scid_1), GSM27453 (NOD.scid1) and GSM27456 (NOD1) in all the analyses presented in this paper. In the source mouse model experiments [4], RNA hybridization was done on Affymetrix gene chip platform MGU74AV2.

### Edge and node weights

The color coding algorithm used in [8] was not suitable for detecting paths in phenotypic context, since they did not have any phenotypic weights. Their weights were solely based on reliabilities of interactions. We modified the color coding algorithm so that it works in phenotypic manner by assigning weights to nodes. We did the weight assignment for each mouse model comparison separately. In order to find the up-regulated paths, we assigned case-control ratios. And to find down-regulated paths, we assigned control-case ratios as weights to nodes. We can thus use the color coding algorithm to find maximum paths in both cases.

We assigned equal weights of 1.0 to all edges from MINT, DIP, KEGG and TransPath, while the edges from BIND were set to 0.33, reflecting large database size of BIND and its reliability of interactions [39].

### Path scoring

The path score is computed as follows. In order to give high penalty for a cascade of unreliable edges, we first multiply all edge weights. In order to reward inclusion of high weight nodes, we sum up all node weights. In the end, we multiply the edge product and the node sum. More precisely, the path scoring scheme is presented in Figure S10 and Formulas (1)–(3) below. We thus move forward on a path by selecting a node and edge so that the total weight is maximized. However, we are not allowed to move forward to a node if its color is inside the sliding window (read more in the next paragraph).
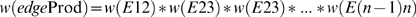
(1)

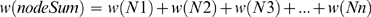
(2)

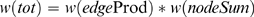
(3)


We used a color coding algorithm for detecting optimal paths [7]. The basic idea of this algorithm is to assign colors (*i.e*., integers) to nodes randomly and detect paths which do not contain same color twice. The restriction on colors guarantees that the detected path is a simple path. When the network is very large, the applicability of this algorithm is challenged by the large computer memory requirements. To address this limitation, we extended the algorithm by using a sliding window so that the distinct color requirement applies only to nodes that are inside the window (Figure S1). That is, unlike the original algorithm which allows no two nodes in a path to have the same color, our algorithm allows no two nodes within the length of the sliding window to have the same color. We first tried to detect a path by using a window length that is equal to the length of detected path. If we did not find a path, we decreased the window length by 1 until we found a path or the window length became 1. This modification improves the performance because it avoids storing of the whole path in computer memory. The algorithm is thus faster and it is capable of detecting longer paths. It is thus more applicable to integrated networks that are usually very large. However, in principle the original version could be used in integrated networks, but it is more probable that there appear memory problems.

### Statistical significance of a path

In order to test for the null hypothesis that the detected path is obtained by chance, we calculated the *p*-values. In order to calculate one *p*-value, we shuffled node and edge weights 10,000 times. For the purpose of computational efficiency, we first tested how promising the *p*-value looks after each shuffle based on the pre-specified cutoff criterion (*p*-value <0.025), then jumped into the next path if the criterion was not met. The full algorithm for the *p*-value calculation is described in the Supplementary Text S1.

### Network harvesting

A network is considered *harvested* if all optimal paths in the network are detected. However, there is not any rigorous way to define when the network is *harvested*, so we took a heuristic approach by assuming that the network is harvested if we come up with 50 consecutive iterations in which the detected path is previously detected. However, since the *p*-value calculation for an optimal path is computationally expensive, we also limited ourselves to finding at most two optimal paths of the same length in each network (*i.e.*, in each mouse model comparison). It is easy to increase this number of paths if required. The algorithm is described in the Supplementary Text S2.

### Characterization of paths

We used a hypergeometric test to identify gene sets from the MSigDB [19] that are over-represented in the molecular paths detected by the EMPath method. First, as a quality control criterion, we restricted the searches to gene sets compiled from pathway databases KEGG, BioCarta, GenMAPP, and GO. Next, we defined the *Gene Symbol Universe* by taking the union of all genes in the selected gene sets. Next, we translated the Swissprot accession numbers of protein nodes of the molecular paths to the Gene Symbols of their encoding genes. These translations are done using Affymetrix annotations of the mouse gene chip platform MGU74Av2, the platform used for NOD mice gene expression experiments. Finally, by using the function *phyper* of the R *stats* package [40] we tested for enrichment of each gene set in each molecular path. In order to account for multiple comparisons, the Benjamini and Hochberg's method for controlling the false discovery rate was applied [41].

### Gene Set Enrichment Analysis

We performed Gene Set Enrichment Analysis (GSEA) of the T1D gene expression data [4] using Java desktop version of the software (February 2006 release). We performed GSEA separately for the two selected phenotype comparisons. Since there was only one sample per phenotype, giving one gene expression value per gene per phenotype, we used the *ratio of classes* statistic of the GSEA for ranking genes. We accessed the gene sets defined in the MSigDB [19] and annotations for the Affymetrix gene chip platform MGU74AV2 via ftp pages of GSEA from within the software interface. The GSEA statistics were computed using 5,000 gene set permutations.

### T1DBase Meta Analysis

First, we selected proteins from the paths detected by EmPath (Figures S2–[Supplementary-material pone.0007323.s012] [Supplementary-material pone.0007323.s013] S5). They were annotated by Uniprot identifiers. We then used EMBL database to find EMBL identifiers for corresponding genes. The NCBI Entrez gene database (http://www.ncbi.nlm.nih.gov/sites/entrez?db=gene) was then searched to find Entrez gene identifiers for those genes. We used these identifiers on the web user interface of the T1DBase Meta analysis tool (http://www.t1dbase.org/page/MetaHome). We performed the expression comparison by using all studies that were available in the Beta Cell Biology Consortium.

## Supporting Information

Text S1 The algorithm for calculating significance of optimal paths detected by EMPath method (p-value calculation).
(0.04 MB DOC)

Text S2 Network harvesting algorithm.
(0.03 MB DOC)

Table S1 Genes found in downregulated paths in insulitis.
(0.04 MB DOC)

Table S2 Significantly enriched pathways in insulitis and type 1 diabetes as derived from detected paths.
(1.06 MB DOC)

Table S3 Enriched upregulated pathways in insulitis.
(0.03 MB DOC)

Table S4 Enriched downregulated pathways in insulitis.
(0.09 MB DOC)

Table S5 Enriched upregulated pathways in type 1 diabetes.
(0.05 MB DOC)

Table S6 Enriched downregulated pathways in type 1 diabetes.
(0.08 MB DOC)

Table S7 Excluded cofactors.
(0.08 MB DOC)

Figure S1 Use of a sliding window to optimize the path detection. The distinct color requirement applies only inside the window. We therefore do not need store the whole path in memory, which makes the detection process faster. In this figure we have an example in which our window size is 2. Our path detection is at a stage in which we have traversed from A- to B to C. And we have {2,3} in denied colors. We can thus continue to either D or E.
(1.00 MB EPS)

Figure S2 Upregulated paths in BDC2.5/NOD vs. NOD comparison. The nodes are colored using the same color code as in Figure 2. Edge annotations related to the source database: K, KEGG; M, MINT.
(1.30 MB EPS)

Figure S3 Downregulated paths in BDC2.5/NOD vs. NOD comparison. The nodes are colored using the same color code as in Figure 2. Edge annotations related to the source database: K, KEGG; M, MINT.
(1.30 MB EPS)

Figure S4 Upregulated paths in BDC2.5/NOD.scid vs. NOD.scid comparison. The nodes are colored using the same color code as in Figure 2. Edge annotations related to the source database: K, KEGG; M, MINT.
(1.30 MB EPS)

Figure S5 Downregulated paths in BDC2.5/NOD.scid vs. NOD.scid comparison. The nodes are colored using the same color code as in Figure 2. Edge annotations related to the source database: K, KEGG; M, MINT.
(1.26 MB EPS)

Figure S6 Meta-analysis for upregulated genes in BDC2.5/NOD vs. NOD comparison. Genes are presented as rows and study group comparisons as columns.
(1.87 MB EPS)

Figure S7 Meta-analysis for downregulated genes in BDC2.5/NOD vs. NOD comparison. Genes are presented as rows and study group comparisons as columns.
(1.86 MB EPS)

Figure S8 Meta-analysis for upregulated genes in BDC2.5/NOD.scid vs. NOD.scid comparison. Genes are presented as rows and study group comparisons as columns.
(2.25 MB EPS)

Figure S9 Meta-analysis for downregulated genes in BDC2.5/NOD.scid vs. NOD.scid comparison. Genes are presented as rows and study comparisons as columns.
(1.67 MB EPS)

Figure S10 Path scoring method. In order to calculate the score for the path, the edge weights are multiplied. All node weights are then summed up. In the end, the edge product and the node sum are multiplied. The total path score is thus (w(E12)* w(E23)*..* w((n-1)N)))*(W(N1)+ W(N2)+..+ W(Nn)).
(1.00 MB EPS)
