# Supplementary material for: Detection of Molecular Paths Associated with Insulitis and Type 1 Diabetes in Non-Obese Diabetic Mouse
Source: PLoS One. 2009 Oct 2;4(10):e7323. doi: 10.1371/journal.pone.0007323 (PMC2749452; doi:10.1371/journal.pone.0007323)
Supplement: Text S1 — The algorithm for calculating significance of optimal paths detected by EMPath method (p-value calculation). (0.04 MB DOC) [file pone.0007323.s001.doc]

**Input**
Graph $G_{Input}$ = ($N_{Input}$,$E_{Input}$,)
Path length k

**Output**
Maximum path of length k
P-value

**Algorithm**
double maxPathScore = detectMaxPath(G, k);
if (maxPathScore > 0) {
 int noMaxRandTrials = 0;
 for (int i = 1; i < = 10 000; i++) {
 Construct $G_{Rand}$ = ($N_{Rand}$,$E_{Rand}$,) by shuffling weights of $N_{Input}$ and $E_{Input}$.

 maxRandPathScore = detectMaxPath($G_{Rand}$, k);
 if (maxRandPathScore >= maxPathScore) {
 noMaxRandTrials++;
 }
 Test if p-value looks promising *
 if (p-value does not look promising) {
 break;
 }
 }
 pValue = noMaxRandTrials / 10 000;
}

function double detectMaxPath(Graph G, int k) {
 for (int = k; i = 0; i--) {
 Try to detect a maximum path of length k in G by using color coding with noColors=i and windowSize=i.
 if (path detected) {
 maxPathScore = score of the detected path
 return maxPathScore;
 }
 }
 return 0;
}

* We test if p-value looks promising by using the following heuristic.

$t$ = Number of permutations
 $k$ = Number of permutations in which the path in random network was more optimal than the path in the original graph
Confidence interval $\approx\left[\frac{k}{t}-\frac{\sqrt{k}}{t}, \frac{k}{t}+\frac{\sqrt{k}}{t}\right]$
if $k-\sqrt{k}>\frac{t}{40}\Rightarrow$ *p*-value not promising
