# Supplementary material for: Detection of Molecular Paths Associated with Insulitis and Type 1 Diabetes in Non-Obese Diabetic Mouse
Source: PLoS One. 2009 Oct 2;4(10):e7323. doi: 10.1371/journal.pone.0007323 (PMC2749452; doi:10.1371/journal.pone.0007323)
Supplement: Text S2 — Network harvesting algorithm. (0.03 MB DOC) [file pone.0007323.s002.doc]

for each case-control combination {
 int noConsecutiveStatSignPaths=0;
 int noStatSignPaths=0;
 S = a set of detected statistically significant paths
 Initialize S to be an empty set
 while (noConsecutiveStatSignPaths < 50 and noStatSignPaths < 2) {
 Try to detect a maximum path in the network.
 Let us denote it maxPath.
 if (no path detected or maxPath is not statistically significant) {
 continue;
 }
 if (S contains maxPath) {
 noConsecutiveStatSignPaths++;
 } else {
 noConsecutiveStatSignPaths=0;
 Insert maxPath into S.
 noStatSignPaths++;
 }
 }
}
