# Supplementary material for: Detection of Molecular Paths Associated with Insulitis and Type 1 Diabetes in Non-Obese Diabetic Mouse
Source: PLoS One. 2009 Oct 2;4(10):e7323. doi: 10.1371/journal.pone.0007323 (PMC2749452; doi:10.1371/journal.pone.0007323)
Supplement: Table S1 — Genes found in downregulated paths in insulitis. (0.04 MB DOC) [file pone.0007323.s003.doc]

| **UniProt ID** | **Name** |
| --- | --- |
| O35400 | Sulfotransferase family cytosolic 2B member 1 |
| P11152 | Lipoprotein lipase precursor |
| P49407 | Beta-arrestin-1 |
| P50428 | Arylsulfatase A precursor |
| P97742 | Carnitine O-palmitoyltransferase I, liver isoform (CPT1) |
| P97813 | Phospholipase D2 |
| P98192 | Dihydroxyacetone phosphate acyltransferase (DHAP-AT) |
| Q5BKQ4 | Pancreatic lipase-related protein 1 precursor |
| Q61469 | Lipid phosphate phosphohydrolase 1 |
| Q6NS52 | Diacylglycerol kinase beta |
| Q8BGL3 | Adult male aorta and vein cDNA, RIKEN full-length enriched library, clone:A530080C09 product |
| Q8VCT4 | Carboxylesterase 3 precursor |
| Q91VR8 | RIKEN cDNA 6720456B07 gene |
| Q99JY8 | Lipid phosphate phosphohydrolase 3 |
| Q9DBF1 | Aldehyde dehydrogenase family 7 member A1 |
| Q9EPR2 | Group XIIA secretory phospholipase A2 precursor |
| Q9JLJ2 | 4-trimethylaminobutyraldehyde dehydrogenase (TMABADH) |
| Q9QUL3 | Group IIE secretory phospholipase A2 precursor |
| Q9R1E6 | Ectonucleotide pyrophosphatase/phosphodiesterase 2 |
| Q9WV54 | Acid ceramidase precursor |
| Q9Z2A7 | Diacylglycerol O-acyltransferase 1 (DGAT1) |
| Q9Z2C4 | Myotubularin-related protein 1 |
