# Supplementary material for: Detection of Molecular Paths Associated with Insulitis and Type 1 Diabetes in Non-Obese Diabetic Mouse
Source: PLoS One. 2009 Oct 2;4(10):e7323. doi: 10.1371/journal.pone.0007323 (PMC2749452; doi:10.1371/journal.pone.0007323)
Supplement: Table S2 — Significantly enriched pathways in insulitis and type 1 diabetes as derived from detected paths. (1.06 MB DOC) [file pone.0007323.s004.doc]

| **Path #** | **Gene set** | **Source** | **n(P&G)** | **n(G)** | **Nominal**  ***p*-value** | **FDR**  ***q*-value** |
| --- | --- | --- | --- | --- | --- | --- |
|  |  |  |  |  |  |  |
| **Enriched in upregulated paths (BDC2.5/NOD *vs.* NOD)** | | | | | | |
| 7 | PTDINSPATHWAY | BioCarta | 3 | 19 | 0.000004 | 0.000103 |
| 7 | HSA00051_FRUCTOSE_AND_MANNOSE_METABOLISM | KEGG | 3 | 27 | 0.000012 | 0.000155 |
| 14 | HSA00530_AMINOSUGARS_METABOLISM | KEGG | 2 | 16 | 0.000025 | 0.000280 |
| 14 | HSA00051_FRUCTOSE_AND_MANNOSE_METABOLISM | KEGG | 2 | 27 | 0.000075 | 0.000410 |
| 7 | FRUCTOSE_AND_MANNOSE_METABOLISM | GenMAPP | 2 | 18 | 0.000481 | 0.003099 |
| 7 | GALACTOSE_METABOLISM | GenMAPP | 2 | 20 | 0.000596 | 0.003099 |
| 7 | HSA00530_AMINOSUGARS_METABOLISM | KEGG | 2 | 16 | 0.000378 | 0.003099 |
| 7 | HSA00052_GALACTOSE_METABOLISM | KEGG | 2 | 24 | 0.000863 | 0.003738 |
| 7 | GLUCONEOGENESIS | GenMAPP | 2 | 39 | 0.002286 | 0.006604 |
| 7 | GLYCOLYSIS | GenMAPP | 2 | 39 | 0.002286 | 0.006604 |
| 7 | GLYCOLYSIS_AND_GLUCONEOGENESIS | GenMAPP | 2 | 35 | 0.001842 | 0.006604 |
| 7 | HSA00010_GLYCOLYSIS_AND_GLUCONEOGENESIS | KEGG | 2 | 45 | 0.003038 | 0.007899 |
| 1 | HSA04630_JAK_STAT_SIGNALING_PATHWAY | KEGG | 4 | 100 | 0.000118 | 0.008023 |
| 11 | HSA04664_FC_EPSILON_RI_SIGNALING_PATHWAY | KEGG | 3 | 62 | 0.000150 | 0.008426 |
| 7 | HSA04664_FC_EPSILON_RI_SIGNALING_PATHWAY | KEGG | 2 | 62 | 0.005717 | 0.013514 |
| 11 | PTDINSPATHWAY | BioCarta | 2 | 19 | 0.000537 | 0.015031 |
| 10 | HSA04664_FC_EPSILON_RI_SIGNALING_PATHWAY | KEGG | 3 | 62 | 0.000260 | 0.015051 |
| 11 | GHPATHWAY | BioCarta | 2 | 24 | 0.000863 | 0.016104 |
| 11 | BCRPATHWAY | BioCarta | 2 | 32 | 0.001540 | 0.017192 |
| 11 | FCER1PATHWAY | BioCarta | 2 | 35 | 0.001842 | 0.017192 |
| 11 | IL2RBPATHWAY | BioCarta | 2 | 34 | 0.001738 | 0.017192 |
| 10 | PTDINSPATHWAY | BioCarta | 2 | 19 | 0.000749 | 0.021715 |
| 10 | GHPATHWAY | BioCarta | 2 | 24 | 0.001202 | 0.023239 |
| 10 | BCRPATHWAY | BioCarta | 2 | 32 | 0.002141 | 0.024750 |
| 10 | FCER1PATHWAY | BioCarta | 2 | 35 | 0.002560 | 0.024750 |
| 10 | IL2RBPATHWAY | BioCarta | 2 | 34 | 0.002417 | 0.024750 |
| 11 | HSA04662_B_CELL_RECEPTOR_SIGNALING_PATHWAY | KEGG | 2 | 47 | 0.003312 | 0.026493 |
| 14 | FRUCTOSE_AND_MANNOSE_METABOLISM | GenMAPP | 1 | 18 | 0.011698 | 0.027784 |
| 14 | GALACTOSE_METABOLISM | GenMAPP | 1 | 20 | 0.012993 | 0.027784 |
| 14 | GLUCONEOGENESIS | GenMAPP | 1 | 39 | 0.025258 | 0.027784 |
| 14 | GLYCOLYSIS | GenMAPP | 1 | 39 | 0.025258 | 0.027784 |
| 14 | GLYCOLYSIS_AND_GLUCONEOGENESIS | GenMAPP | 1 | 35 | 0.022682 | 0.027784 |
| 14 | HSA00052_GALACTOSE_METABOLISM | KEGG | 1 | 24 | 0.015582 | 0.027784 |
| 14 | HSA00500_STARCH_AND_SUCROSE_METABOLISM | KEGG | 1 | 37 | 0.023971 | 0.027784 |
| 14 | STARCH_AND_SUCROSE_METABOLISM | GenMAPP | 1 | 18 | 0.011698 | 0.027784 |
| 14 | HSA00010_GLYCOLYSIS_AND_GLUCONEOGENESIS | KEGG | 1 | 45 | 0.029115 | 0.029115 |
| 1 | ERKPATHWAY | BioCarta | 2 | 25 | 0.002215 | 0.037656 |
| 1 | IL2PATHWAY | BioCarta | 2 | 22 | 0.001713 | 0.037656 |
| 1 | IL6PATHWAY | BioCarta | 2 | 20 | 0.001414 | 0.037656 |
| 10 | HSA04662_B_CELL_RECEPTOR_SIGNALING_PATHWAY | KEGG | 2 | 47 | 0.004591 | 0.038041 |
| 3 | BCRPATHWAY | BioCarta | 2 | 32 | 0.001540 | 0.039910 |
| 3 | ERKPATHWAY | BioCarta | 2 | 25 | 0.000937 | 0.039910 |
| 3 | FCER1PATHWAY | BioCarta | 2 | 35 | 0.001842 | 0.039910 |
| 1 | BCRPATHWAY | BioCarta | 2 | 32 | 0.003623 | 0.042031 |
| 1 | FCER1PATHWAY | BioCarta | 2 | 35 | 0.004327 | 0.042031 |
| 1 | IL2RBPATHWAY | BioCarta | 2 | 34 | 0.004086 | 0.042031 |
| 11 | INTEGRIN_MEDIATED_CELL_ADHESION_KEGG | GenMAPP | 2 | 68 | 0.006851 | 0.047960 |
| **Enriched in downregulated paths (BDC2.5/NOD *vs.* NOD)** | | | | | | |
| 11 | GLYCEROLIPID_METABOLISM | GenMAPP | 3 | 24 | 0.000000 | 0.000011 |
| 11 | HSA00561_GLYCEROLIPID_METABOLISM | KEGG | 3 | 28 | 0.000001 | 0.000011 |
| 4 | GLYCEROLIPID_METABOLISM | GenMAPP | 3 | 24 | 0.000000 | 0.000011 |
| 4 | HSA00561_GLYCEROLIPID_METABOLISM | KEGG | 3 | 28 | 0.000001 | 0.000011 |
| 6 | GLYCEROLIPID_METABOLISM | GenMAPP | 3 | 24 | 0.000000 | 0.000011 |
| 6 | HSA00561_GLYCEROLIPID_METABOLISM | KEGG | 3 | 28 | 0.000001 | 0.000011 |
| 10 | GLYCEROLIPID_METABOLISM | GenMAPP | 3 | 24 | 0.000002 | 0.000015 |
| 10 | HSA00561_GLYCEROLIPID_METABOLISM | KEGG | 3 | 28 | 0.000003 | 0.000015 |
| 2 | GLYCEROLIPID_METABOLISM | GenMAPP | 3 | 24 | 0.000002 | 0.000043 |
| 2 | HSA00561_GLYCEROLIPID_METABOLISM | KEGG | 3 | 28 | 0.000003 | 0.000043 |
| 1 | GLYCEROLIPID_METABOLISM | GenMAPP | 3 | 24 | 0.000002 | 0.000045 |
| 1 | HSA00561_GLYCEROLIPID_METABOLISM | KEGG | 3 | 28 | 0.000003 | 0.000045 |
| 9 | GLYCEROLIPID_METABOLISM | GenMAPP | 3 | 24 | 0.000004 | 0.000064 |
| 9 | HSA00561_GLYCEROLIPID_METABOLISM | KEGG | 3 | 28 | 0.000007 | 0.000064 |
| 12 | GLYCEROLIPID_METABOLISM | GenMAPP | 3 | 24 | 0.000004 | 0.000111 |
| 12 | HSA00561_GLYCEROLIPID_METABOLISM | KEGG | 3 | 28 | 0.000007 | 0.000111 |
| 10 | STATIN_PATHWAY_PHARMGKB | GenMAPP | 2 | 16 | 0.000152 | 0.000557 |
| 9 | STATIN_PATHWAY_PHARMGKB | GenMAPP | 2 | 16 | 0.000253 | 0.001600 |
| 9 | HSA00565_ETHER_LIPID_METABOLISM | KEGG | 2 | 21 | 0.000441 | 0.002093 |
| 8 | HSA00071_FATTY_ACID_METABOLISM | KEGG | 2 | 29 | 0.000847 | 0.002311 |
| 8 | HSA00120_BILE_ACID_BIOSYNTHESIS | KEGG | 2 | 20 | 0.000399 | 0.002311 |
| 8 | HSA00220_UREA_CYCLE_AND_METABOLISM_OF_AMINO_GROUPS | KEGG | 2 | 21 | 0.000441 | 0.002311 |
| 8 | HSA00310_LYSINE_DEGRADATION | KEGG | 2 | 29 | 0.000847 | 0.002311 |
| 8 | HSA00340_HISTIDINE_METABOLISM | KEGG | 2 | 19 | 0.000359 | 0.002311 |
| 8 | HSA00410_BETA_ALANINE_METABOLISM | KEGG | 2 | 17 | 0.000286 | 0.002311 |
| 8 | HSA00561_GLYCEROLIPID_METABOLISM | KEGG | 2 | 28 | 0.000789 | 0.002311 |
| 8 | HSA00620_PYRUVATE_METABOLISM | KEGG | 2 | 28 | 0.000789 | 0.002311 |
| 8 | HSA00640_PROPANOATE_METABOLISM | KEGG | 2 | 23 | 0.000530 | 0.002311 |
| 8 | HSA00650_BUTANOATE_METABOLISM | KEGG | 2 | 28 | 0.000789 | 0.002311 |
| 8 | HSA00903_LIMONENE_AND_PINENE_DEGRADATION | KEGG | 2 | 17 | 0.000286 | 0.002311 |
| 8 | HSA00280_VALINE_LEUCINE_AND_ISOLEUCINE_DEGRADATION | KEGG | 2 | 33 | 0.001099 | 0.002693 |
| 8 | HSA00380_TRYPTOPHAN_METABOLISM | KEGG | 2 | 34 | 0.001167 | 0.002693 |
| 1 | HSA00565_ETHER_LIPID_METABOLISM | KEGG | 2 | 21 | 0.000265 | 0.002920 |
| 8 | HSA00010_GLYCOLYSIS_AND_GLUCONEOGENESIS | KEGG | 2 | 45 | 0.002045 | 0.004381 |
| 5 | GLYCEROLIPID_METABOLISM | GenMAPP | 2 | 24 | 0.000348 | 0.005284 |
| 5 | HSA00071_FATTY_ACID_METABOLISM | KEGG | 2 | 29 | 0.000511 | 0.005284 |
| 5 | HSA00561_GLYCEROLIPID_METABOLISM | KEGG | 2 | 28 | 0.000476 | 0.005284 |
| 9 | HSA00564_GLYCEROPHOSPHOLIPID_METABOLISM | KEGG | 2 | 38 | 0.001458 | 0.005542 |
| 3 | GLYCEROLIPID_METABOLISM | GenMAPP | 2 | 24 | 0.000348 | 0.005796 |
| 3 | HSA00071_FATTY_ACID_METABOLISM | KEGG | 2 | 29 | 0.000511 | 0.005796 |
| 3 | HSA00561_GLYCEROLIPID_METABOLISM | KEGG | 2 | 28 | 0.000476 | 0.005796 |
| 1 | HSA00564_GLYCEROPHOSPHOLIPID_METABOLISM | KEGG | 2 | 38 | 0.000882 | 0.007276 |
| 9 | HSA03320_PPAR_SIGNALING_PATHWAY | KEGG | 2 | 50 | 0.002522 | 0.007985 |
| 3 | HSA03320_PPAR_SIGNALING_PATHWAY | KEGG | 2 | 50 | 0.001529 | 0.012995 |
| 11 | BETA_ALANINE_METABOLISM | GenMAPP | 1 | 17 | 0.016531 | 0.037453 |
| 11 | BILE_ACID_BIOSYNTHESIS | GenMAPP | 1 | 15 | 0.014596 | 0.037453 |
| 11 | BUTANOATE_METABOLISM | GenMAPP | 1 | 19 | 0.018464 | 0.037453 |
| 11 | GLYCEROPHOSPHOLIPID_METABOLISM | GenMAPP | 1 | 28 | 0.027130 | 0.037453 |
| 11 | HISTIDINE_METABOLISM | GenMAPP | 1 | 15 | 0.014596 | 0.037453 |
| 11 | HSA00071_FATTY_ACID_METABOLISM | KEGG | 1 | 29 | 0.028090 | 0.037453 |
| 11 | HSA00120_BILE_ACID_BIOSYNTHESIS | KEGG | 1 | 20 | 0.019430 | 0.037453 |
| 11 | HSA00220_UREA_CYCLE_AND_METABOLISM_OF_AMINO_GROUPS | KEGG | 1 | 21 | 0.020394 | 0.037453 |
| 11 | HSA00310_LYSINE_DEGRADATION | KEGG | 1 | 29 | 0.028090 | 0.037453 |
| 11 | HSA00340_HISTIDINE_METABOLISM | KEGG | 1 | 19 | 0.018464 | 0.037453 |
| 11 | HSA00410_BETA_ALANINE_METABOLISM | KEGG | 1 | 17 | 0.016531 | 0.037453 |
| 11 | HSA00565_ETHER_LIPID_METABOLISM | KEGG | 1 | 21 | 0.020394 | 0.037453 |
| 11 | HSA00600_SPHINGOLIPID_METABOLISM | KEGG | 1 | 20 | 0.019430 | 0.037453 |
| 11 | HSA00620_PYRUVATE_METABOLISM | KEGG | 1 | 28 | 0.027130 | 0.037453 |
| 11 | HSA00640_PROPANOATE_METABOLISM | KEGG | 1 | 23 | 0.022322 | 0.037453 |
| 11 | HSA00650_BUTANOATE_METABOLISM | KEGG | 1 | 28 | 0.027130 | 0.037453 |
| 11 | HSA00903_LIMONENE_AND_PINENE_DEGRADATION | KEGG | 1 | 17 | 0.016531 | 0.037453 |
| 11 | LYSINE_DEGRADATION | GenMAPP | 1 | 18 | 0.017498 | 0.037453 |
| 11 | PROPANOATE_METABOLISM | GenMAPP | 1 | 23 | 0.022322 | 0.037453 |
| 11 | PYRUVATE_METABOLISM | GenMAPP | 1 | 27 | 0.026170 | 0.037453 |
| 11 | TRYPTOPHAN_METABOLISM | GenMAPP | 1 | 29 | 0.028090 | 0.037453 |
| 11 | VALINE_LEUCINE_AND_ISOLEUCINE_DEGRADATION | GenMAPP | 1 | 26 | 0.025209 | 0.037453 |
| 4 | BETA_ALANINE_METABOLISM | GenMAPP | 1 | 17 | 0.016531 | 0.037453 |
| 4 | BILE_ACID_BIOSYNTHESIS | GenMAPP | 1 | 15 | 0.014596 | 0.037453 |
| 4 | BUTANOATE_METABOLISM | GenMAPP | 1 | 19 | 0.018464 | 0.037453 |
| 4 | GLYCEROPHOSPHOLIPID_METABOLISM | GenMAPP | 1 | 28 | 0.027130 | 0.037453 |
| 4 | HISTIDINE_METABOLISM | GenMAPP | 1 | 15 | 0.014596 | 0.037453 |
| 4 | HSA00071_FATTY_ACID_METABOLISM | KEGG | 1 | 29 | 0.028090 | 0.037453 |
| 4 | HSA00120_BILE_ACID_BIOSYNTHESIS | KEGG | 1 | 20 | 0.019430 | 0.037453 |
| 4 | HSA00220_UREA_CYCLE_AND_METABOLISM_OF_AMINO_GROUPS | KEGG | 1 | 21 | 0.020394 | 0.037453 |
| 4 | HSA00310_LYSINE_DEGRADATION | KEGG | 1 | 29 | 0.028090 | 0.037453 |
| 4 | HSA00340_HISTIDINE_METABOLISM | KEGG | 1 | 19 | 0.018464 | 0.037453 |
| 4 | HSA00410_BETA_ALANINE_METABOLISM | KEGG | 1 | 17 | 0.016531 | 0.037453 |
| 4 | HSA00565_ETHER_LIPID_METABOLISM | KEGG | 1 | 21 | 0.020394 | 0.037453 |
| 4 | HSA00600_SPHINGOLIPID_METABOLISM | KEGG | 1 | 20 | 0.019430 | 0.037453 |
| 4 | HSA00620_PYRUVATE_METABOLISM | KEGG | 1 | 28 | 0.027130 | 0.037453 |
| 4 | HSA00640_PROPANOATE_METABOLISM | KEGG | 1 | 23 | 0.022322 | 0.037453 |
| 4 | HSA00650_BUTANOATE_METABOLISM | KEGG | 1 | 28 | 0.027130 | 0.037453 |
| 4 | HSA00903_LIMONENE_AND_PINENE_DEGRADATION | KEGG | 1 | 17 | 0.016531 | 0.037453 |
| 4 | LYSINE_DEGRADATION | GenMAPP | 1 | 18 | 0.017498 | 0.037453 |
| 4 | PROPANOATE_METABOLISM | GenMAPP | 1 | 23 | 0.022322 | 0.037453 |
| 4 | PYRUVATE_METABOLISM | GenMAPP | 1 | 27 | 0.026170 | 0.037453 |
| 4 | TRYPTOPHAN_METABOLISM | GenMAPP | 1 | 29 | 0.028090 | 0.037453 |
| 4 | VALINE_LEUCINE_AND_ISOLEUCINE_DEGRADATION | GenMAPP | 1 | 26 | 0.025209 | 0.037453 |
| 6 | BETA_ALANINE_METABOLISM | GenMAPP | 1 | 17 | 0.016531 | 0.037453 |
| 6 | BILE_ACID_BIOSYNTHESIS | GenMAPP | 1 | 15 | 0.014596 | 0.037453 |
| 6 | BUTANOATE_METABOLISM | GenMAPP | 1 | 19 | 0.018464 | 0.037453 |
| 6 | GLYCEROPHOSPHOLIPID_METABOLISM | GenMAPP | 1 | 28 | 0.027130 | 0.037453 |
| 6 | HISTIDINE_METABOLISM | GenMAPP | 1 | 15 | 0.014596 | 0.037453 |
| 6 | HSA00071_FATTY_ACID_METABOLISM | KEGG | 1 | 29 | 0.028090 | 0.037453 |
| 6 | HSA00120_BILE_ACID_BIOSYNTHESIS | KEGG | 1 | 20 | 0.019430 | 0.037453 |
| 6 | HSA00220_UREA_CYCLE_AND_METABOLISM_OF_AMINO_GROUPS | KEGG | 1 | 21 | 0.020394 | 0.037453 |
| 6 | HSA00310_LYSINE_DEGRADATION | KEGG | 1 | 29 | 0.028090 | 0.037453 |
| 6 | HSA00340_HISTIDINE_METABOLISM | KEGG | 1 | 19 | 0.018464 | 0.037453 |
| 6 | HSA00410_BETA_ALANINE_METABOLISM | KEGG | 1 | 17 | 0.016531 | 0.037453 |
| 6 | HSA00565_ETHER_LIPID_METABOLISM | KEGG | 1 | 21 | 0.020394 | 0.037453 |
| 6 | HSA00600_SPHINGOLIPID_METABOLISM | KEGG | 1 | 20 | 0.019430 | 0.037453 |
| 6 | HSA00620_PYRUVATE_METABOLISM | KEGG | 1 | 28 | 0.027130 | 0.037453 |
| 6 | HSA00640_PROPANOATE_METABOLISM | KEGG | 1 | 23 | 0.022322 | 0.037453 |
| 6 | HSA00650_BUTANOATE_METABOLISM | KEGG | 1 | 28 | 0.027130 | 0.037453 |
| 6 | HSA00903_LIMONENE_AND_PINENE_DEGRADATION | KEGG | 1 | 17 | 0.016531 | 0.037453 |
| 6 | LYSINE_DEGRADATION | GenMAPP | 1 | 18 | 0.017498 | 0.037453 |
| 6 | PROPANOATE_METABOLISM | GenMAPP | 1 | 23 | 0.022322 | 0.037453 |
| 6 | PYRUVATE_METABOLISM | GenMAPP | 1 | 27 | 0.026170 | 0.037453 |
| 6 | TRYPTOPHAN_METABOLISM | GenMAPP | 1 | 29 | 0.028090 | 0.037453 |
| 6 | VALINE_LEUCINE_AND_ISOLEUCINE_DEGRADATION | GenMAPP | 1 | 26 | 0.025209 | 0.037453 |
| 11 | ARGININE_AND_PROLINE_METABOLISM | GenMAPP | 1 | 32 | 0.030966 | 0.038968 |
| 11 | HSA00280_VALINE_LEUCINE_AND_ISOLEUCINE_DEGRADATION | KEGG | 1 | 33 | 0.031923 | 0.038968 |
| 11 | HSA00380_TRYPTOPHAN_METABOLISM | KEGG | 1 | 34 | 0.032879 | 0.038968 |
| 4 | ARGININE_AND_PROLINE_METABOLISM | GenMAPP | 1 | 32 | 0.030966 | 0.038968 |
| 4 | HSA00280_VALINE_LEUCINE_AND_ISOLEUCINE_DEGRADATION | KEGG | 1 | 33 | 0.031923 | 0.038968 |
| 4 | HSA00380_TRYPTOPHAN_METABOLISM | KEGG | 1 | 34 | 0.032879 | 0.038968 |
| 6 | ARGININE_AND_PROLINE_METABOLISM | GenMAPP | 1 | 32 | 0.030966 | 0.038968 |
| 6 | HSA00280_VALINE_LEUCINE_AND_ISOLEUCINE_DEGRADATION | KEGG | 1 | 33 | 0.031923 | 0.038968 |
| 6 | HSA00380_TRYPTOPHAN_METABOLISM | KEGG | 1 | 34 | 0.032879 | 0.038968 |
| 11 | GLUCONEOGENESIS | GenMAPP | 1 | 39 | 0.037653 | 0.040163 |
| 11 | GLYCOLYSIS | GenMAPP | 1 | 39 | 0.037653 | 0.040163 |
| 11 | HSA00564_GLYCEROPHOSPHOLIPID_METABOLISM | KEGG | 1 | 38 | 0.036699 | 0.040163 |
| 4 | GLUCONEOGENESIS | GenMAPP | 1 | 39 | 0.037653 | 0.040163 |
| 4 | GLYCOLYSIS | GenMAPP | 1 | 39 | 0.037653 | 0.040163 |
| 4 | HSA00564_GLYCEROPHOSPHOLIPID_METABOLISM | KEGG | 1 | 38 | 0.036699 | 0.040163 |
| 6 | GLUCONEOGENESIS | GenMAPP | 1 | 39 | 0.037653 | 0.040163 |
| 6 | GLYCOLYSIS | GenMAPP | 1 | 39 | 0.037653 | 0.040163 |
| 6 | HSA00564_GLYCEROPHOSPHOLIPID_METABOLISM | KEGG | 1 | 38 | 0.036699 | 0.040163 |
| 11 | HSA00010_GLYCOLYSIS_AND_GLUCONEOGENESIS | KEGG | 1 | 45 | 0.043360 | 0.044759 |
| 4 | HSA00010_GLYCOLYSIS_AND_GLUCONEOGENESIS | KEGG | 1 | 45 | 0.043360 | 0.044759 |
| 6 | HSA00010_GLYCOLYSIS_AND_GLUCONEOGENESIS | KEGG | 1 | 45 | 0.043360 | 0.044759 |
| 8 | BILE_ACID_BIOSYNTHESIS | GenMAPP | 1 | 15 | 0.024216 | 0.045405 |
| 8 | HISTIDINE_METABOLISM | GenMAPP | 1 | 15 | 0.024216 | 0.045405 |
| 11 | CELL_GROWTH_AND_OR_MAINTENANCE | GO | 1 | 50 | 0.048099 | 0.048099 |
| 4 | CELL_GROWTH_AND_OR_MAINTENANCE | GO | 1 | 50 | 0.048099 | 0.048099 |
| 6 | CELL_GROWTH_AND_OR_MAINTENANCE | GO | 1 | 50 | 0.048099 | 0.048099 |
| 8 | BETA_ALANINE_METABOLISM | GenMAPP | 1 | 17 | 0.027409 | 0.048243 |
| 8 | BUTANOATE_METABOLISM | GenMAPP | 1 | 19 | 0.030593 | 0.048243 |
| 8 | HSA00565_ETHER_LIPID_METABOLISM | KEGG | 1 | 21 | 0.033770 | 0.048243 |
| 8 | HSA00600_SPHINGOLIPID_METABOLISM | KEGG | 1 | 20 | 0.032183 | 0.048243 |
| 8 | LYSINE_DEGRADATION | GenMAPP | 1 | 18 | 0.029002 | 0.048243 |
| 1 | BETA_ALANINE_METABOLISM | GenMAPP | 1 | 17 | 0.021984 | 0.049213 |
| 1 | BILE_ACID_BIOSYNTHESIS | GenMAPP | 1 | 15 | 0.019417 | 0.049213 |
| 1 | BUTANOATE_METABOLISM | GenMAPP | 1 | 19 | 0.024547 | 0.049213 |
| 1 | GLYCEROPHOSPHOLIPID_METABOLISM | GenMAPP | 1 | 28 | 0.036015 | 0.049213 |
| 1 | HISTIDINE_METABOLISM | GenMAPP | 1 | 15 | 0.019417 | 0.049213 |
| 1 | HSA00071_FATTY_ACID_METABOLISM | KEGG | 1 | 29 | 0.037283 | 0.049213 |
| 1 | HSA00120_BILE_ACID_BIOSYNTHESIS | KEGG | 1 | 20 | 0.025826 | 0.049213 |
| 1 | HSA00220_UREA_CYCLE_AND_METABOLISM_OF_AMINO_GROUPS | KEGG | 1 | 21 | 0.027104 | 0.049213 |
| 1 | HSA00310_LYSINE_DEGRADATION | KEGG | 1 | 29 | 0.037283 | 0.049213 |
| 1 | HSA00340_HISTIDINE_METABOLISM | KEGG | 1 | 19 | 0.024547 | 0.049213 |
| 1 | HSA00410_BETA_ALANINE_METABOLISM | KEGG | 1 | 17 | 0.021984 | 0.049213 |
| 1 | HSA00600_SPHINGOLIPID_METABOLISM | KEGG | 1 | 20 | 0.025826 | 0.049213 |
| 1 | HSA00620_PYRUVATE_METABOLISM | KEGG | 1 | 28 | 0.036015 | 0.049213 |
| 1 | HSA00640_PROPANOATE_METABOLISM | KEGG | 1 | 23 | 0.029656 | 0.049213 |
| 1 | HSA00650_BUTANOATE_METABOLISM | KEGG | 1 | 28 | 0.036015 | 0.049213 |
| 1 | HSA00903_LIMONENE_AND_PINENE_DEGRADATION | KEGG | 1 | 17 | 0.021984 | 0.049213 |
| 1 | LYSINE_DEGRADATION | GenMAPP | 1 | 18 | 0.023266 | 0.049213 |
| 1 | PROPANOATE_METABOLISM | GenMAPP | 1 | 23 | 0.029656 | 0.049213 |
| 1 | PYRUVATE_METABOLISM | GenMAPP | 1 | 27 | 0.034746 | 0.049213 |
| 1 | TRYPTOPHAN_METABOLISM | GenMAPP | 1 | 29 | 0.037283 | 0.049213 |
| 1 | VALINE_LEUCINE_AND_ISOLEUCINE_DEGRADATION | GenMAPP | 1 | 26 | 0.033475 | 0.049213 |
| 10 | HSA00565_ETHER_LIPID_METABOLISM | KEGG | 1 | 21 | 0.027104 | 0.049691 |
| 10 | HSA00600_SPHINGOLIPID_METABOLISM | KEGG | 1 | 20 | 0.025826 | 0.049691 |
| 10 | HSA05010_ALZHEIMERS_DISEASE | KEGG | 1 | 21 | 0.027104 | 0.049691 |
| 2 | BETA_ALANINE_METABOLISM | GenMAPP | 1 | 17 | 0.021984 | 0.049711 |
| 2 | BILE_ACID_BIOSYNTHESIS | GenMAPP | 1 | 15 | 0.019417 | 0.049711 |
| 2 | BUTANOATE_METABOLISM | GenMAPP | 1 | 19 | 0.024547 | 0.049711 |
| 2 | GLYCEROPHOSPHOLIPID_METABOLISM | GenMAPP | 1 | 28 | 0.036015 | 0.049711 |
| 2 | HISTIDINE_METABOLISM | GenMAPP | 1 | 15 | 0.019417 | 0.049711 |
| 2 | HSA00071_FATTY_ACID_METABOLISM | KEGG | 1 | 29 | 0.037283 | 0.049711 |
| 2 | HSA00120_BILE_ACID_BIOSYNTHESIS | KEGG | 1 | 20 | 0.025826 | 0.049711 |
| 2 | HSA00220_UREA_CYCLE_AND_METABOLISM_OF_AMINO_GROUPS | KEGG | 1 | 21 | 0.027104 | 0.049711 |
| 2 | HSA00310_LYSINE_DEGRADATION | KEGG | 1 | 29 | 0.037283 | 0.049711 |
| 2 | HSA00340_HISTIDINE_METABOLISM | KEGG | 1 | 19 | 0.024547 | 0.049711 |
| 2 | HSA00410_BETA_ALANINE_METABOLISM | KEGG | 1 | 17 | 0.021984 | 0.049711 |
| 2 | HSA00565_ETHER_LIPID_METABOLISM | KEGG | 1 | 21 | 0.027104 | 0.049711 |
| 2 | HSA00600_SPHINGOLIPID_METABOLISM | KEGG | 1 | 20 | 0.025826 | 0.049711 |
| 2 | HSA00620_PYRUVATE_METABOLISM | KEGG | 1 | 28 | 0.036015 | 0.049711 |
| 2 | HSA00640_PROPANOATE_METABOLISM | KEGG | 1 | 23 | 0.029656 | 0.049711 |
| 2 | HSA00650_BUTANOATE_METABOLISM | KEGG | 1 | 28 | 0.036015 | 0.049711 |
| 2 | HSA00903_LIMONENE_AND_PINENE_DEGRADATION | KEGG | 1 | 17 | 0.021984 | 0.049711 |
| 2 | LYSINE_DEGRADATION | GenMAPP | 1 | 18 | 0.023266 | 0.049711 |
| 2 | PROPANOATE_METABOLISM | GenMAPP | 1 | 23 | 0.029656 | 0.049711 |
| 2 | PYRUVATE_METABOLISM | GenMAPP | 1 | 27 | 0.034746 | 0.049711 |
| 2 | TRYPTOPHAN_METABOLISM | GenMAPP | 1 | 29 | 0.037283 | 0.049711 |
| 2 | VALINE_LEUCINE_AND_ISOLEUCINE_DEGRADATION | GenMAPP | 1 | 26 | 0.033475 | 0.049711 |
| **Enriched in upregulated paths (BDC2.5/NOD.scid *vs.* NOD.scid)** | | | | | | |
| 1 | EGFPATHWAY | BioCarta | 4 | 25 | 0.000000 | 0.000040 |
| 3 | HSA04630_JAK_STAT_SIGNALING_PATHWAY | KEGG | 5 | 100 | 0.000002 | 0.000102 |
| 1 | HSA05213_ENDOMETRIAL_CANCER | KEGG | 4 | 42 | 0.000004 | 0.000128 |
| 1 | HSA05223_NON_SMALL_CELL_LUNG_CANCER | KEGG | 4 | 43 | 0.000004 | 0.000128 |
| 1 | CTLA4PATHWAY | BioCarta | 3 | 15 | 0.000008 | 0.000131 |
| 1 | ERK5PATHWAY | BioCarta | 3 | 16 | 0.000010 | 0.000131 |
| 1 | HSA05214_GLIOMA | KEGG | 4 | 50 | 0.000007 | 0.000131 |
| 1 | PTENPATHWAY | BioCarta | 3 | 16 | 0.000010 | 0.000131 |
| 1 | NGFPATHWAY | BioCarta | 3 | 17 | 0.000012 | 0.000140 |
| 1 | IGF1PATHWAY | BioCarta | 3 | 18 | 0.000014 | 0.000149 |
| 1 | INSULINPATHWAY | BioCarta | 3 | 19 | 0.000017 | 0.000159 |
| 1 | GLEEVECPATHWAY | BioCarta | 3 | 20 | 0.000019 | 0.000169 |
| 1 | HSA04012_ERBB_SIGNALING_PATHWAY | KEGG | 4 | 67 | 0.000024 | 0.000178 |
| 1 | HSA05215_PROSTATE_CANCER | KEGG | 4 | 66 | 0.000023 | 0.000178 |
| 1 | CREBPATHWAY | BioCarta | 3 | 22 | 0.000026 | 0.000179 |
| 1 | HSA05210_COLORECTAL_CANCER | KEGG | 4 | 70 | 0.000029 | 0.000180 |
| 1 | TPOPATHWAY | BioCarta | 3 | 23 | 0.000030 | 0.000180 |
| 1 | GHPATHWAY | BioCarta | 3 | 24 | 0.000034 | 0.000193 |
| 1 | PDGFPATHWAY | BioCarta | 3 | 25 | 0.000039 | 0.000207 |
| 3 | EPOPATHWAY | BioCarta | 3 | 19 | 0.000011 | 0.000326 |
| 1 | METPATHWAY | BioCarta | 3 | 30 | 0.000068 | 0.000344 |
| 3 | GHPATHWAY | BioCarta | 3 | 24 | 0.000023 | 0.000451 |
| 1 | IL2RBPATHWAY | BioCarta | 3 | 34 | 0.000100 | 0.000479 |
| 1 | FCER1PATHWAY | BioCarta | 3 | 35 | 0.000109 | 0.000498 |
| 1 | TCRPATHWAY | BioCarta | 3 | 40 | 0.000163 | 0.000713 |
| 2 | GLYCEROLIPID_METABOLISM | GenMAPP | 2 | 24 | 0.000175 | 0.001198 |
| 2 | HSA00561_GLYCEROLIPID_METABOLISM | KEGG | 2 | 28 | 0.000240 | 0.001198 |
| 12 | PTENPATHWAY | BioCarta | 3 | 16 | 0.000014 | 0.001311 |
| 13 | PTENPATHWAY | BioCarta | 3 | 16 | 0.000014 | 0.001311 |
| 14 | PTENPATHWAY | BioCarta | 3 | 16 | 0.000014 | 0.001311 |
| 1 | HSA04510_FOCAL_ADHESION | KEGG | 4 | 137 | 0.000402 | 0.001544 |
| 1 | HSA05211_RENAL_CELL_CARCINOMA | KEGG | 3 | 54 | 0.000402 | 0.001544 |
| 1 | HSA05218_MELANOMA | KEGG | 3 | 54 | 0.000402 | 0.001544 |
| 10 | GLYCEROLIPID_METABOLISM | GenMAPP | 2 | 24 | 0.000348 | 0.001747 |
| 10 | HSA00561_GLYCEROLIPID_METABOLISM | KEGG | 2 | 28 | 0.000476 | 0.001747 |
| 10 | HSA00980_METABOLISM_OF_XENOBIOTICS_BY_CYTOCHROME_P450 | KEGG | 2 | 26 | 0.000410 | 0.001747 |
| 8 | GLYCEROLIPID_METABOLISM | GenMAPP | 2 | 24 | 0.000348 | 0.001747 |
| 8 | HSA00561_GLYCEROLIPID_METABOLISM | KEGG | 2 | 28 | 0.000476 | 0.001747 |
| 8 | HSA00980_METABOLISM_OF_XENOBIOTICS_BY_CYTOCHROME_P450 | KEGG | 2 | 26 | 0.000410 | 0.001747 |
| 7 | PTENPATHWAY | BioCarta | 3 | 16 | 0.000019 | 0.001797 |
| 10 | HSA00380_TRYPTOPHAN_METABOLISM | KEGG | 2 | 34 | 0.000705 | 0.001939 |
| 8 | HSA00380_TRYPTOPHAN_METABOLISM | KEGG | 2 | 34 | 0.000705 | 0.001939 |
| 1 | HSA04664_FC_EPSILON_RI_SIGNALING_PATHWAY | KEGG | 3 | 62 | 0.000605 | 0.002151 |
| 1 | HSA05212_PANCREATIC_CANCER | KEGG | 3 | 62 | 0.000605 | 0.002151 |
| 1 | HSA05220_CHRONIC_MYELOID_LEUKEMIA | KEGG | 3 | 63 | 0.000634 | 0.002175 |
| 1 | AKTPATHWAY | BioCarta | 2 | 15 | 0.000787 | 0.002223 |
| 1 | HCMVPATHWAY | BioCarta | 2 | 15 | 0.000787 | 0.002223 |
| 1 | IGF1MTORPATHWAY | BioCarta | 2 | 15 | 0.000787 | 0.002223 |
| 1 | IL7PATHWAY | BioCarta | 2 | 15 | 0.000787 | 0.002223 |
| 1 | NKCELLSPATHWAY | BioCarta | 2 | 15 | 0.000787 | 0.002223 |
| 1 | SPRYPATHWAY | BioCarta | 2 | 15 | 0.000787 | 0.002223 |
| 1 | GCRPATHWAY | BioCarta | 2 | 16 | 0.000898 | 0.002464 |
| 1 | BADPATHWAY | BioCarta | 2 | 17 | 0.001016 | 0.002508 |
| 1 | HSA04660_T_CELL_RECEPTOR_SIGNALING_PATHWAY | KEGG | 3 | 74 | 0.001019 | 0.002508 |
| 1 | PAR1PATHWAY | BioCarta | 2 | 17 | 0.001016 | 0.002508 |
| 1 | RAC1PATHWAY | BioCarta | 2 | 17 | 0.001016 | 0.002508 |
| 1 | HSA04320_DORSO_VENTRAL_AXIS_FORMATION | KEGG | 2 | 18 | 0.001142 | 0.002740 |
| 1 | ECMPATHWAY | BioCarta | 2 | 19 | 0.001274 | 0.002857 |
| 1 | HSA04650_NATURAL_KILLER_CELL_MEDIATED_CYTOTOXICITY | KEGG | 3 | 80 | 0.001280 | 0.002857 |
| 1 | RACCYCDPATHWAY | BioCarta | 2 | 19 | 0.001274 | 0.002857 |
| 9 | GLYCEROLIPID_METABOLISM | GenMAPP | 2 | 24 | 0.000578 | 0.002894 |
| 9 | HSA00561_GLYCEROLIPID_METABOLISM | KEGG | 2 | 28 | 0.000789 | 0.002894 |
| 9 | HSA00980_METABOLISM_OF_XENOBIOTICS_BY_CYTOCHROME_P450 | KEGG | 2 | 26 | 0.000680 | 0.002894 |
| 1 | MTORPATHWAY | BioCarta | 2 | 20 | 0.001414 | 0.003084 |
| 9 | HSA00380_TRYPTOPHAN_METABOLISM | KEGG | 2 | 34 | 0.001167 | 0.003209 |
| 1 | EDG1PATHWAY | BioCarta | 2 | 22 | 0.001713 | 0.003500 |
| 1 | EIF4PATHWAY | BioCarta | 2 | 22 | 0.001713 | 0.003500 |
| 1 | RASPATHWAY | BioCarta | 2 | 22 | 0.001713 | 0.003500 |
| 1 | CXCR4PATHWAY | BioCarta | 2 | 23 | 0.001874 | 0.003671 |
| 1 | GSK3PATHWAY | BioCarta | 2 | 23 | 0.001874 | 0.003671 |
| 1 | HSA04910_INSULIN_SIGNALING_PATHWAY | KEGG | 3 | 93 | 0.001982 | 0.003806 |
| 1 | VEGFPATHWAY | BioCarta | 2 | 24 | 0.002041 | 0.003842 |
| 1 | ERKPATHWAY | BioCarta | 2 | 25 | 0.002215 | 0.004012 |
| 1 | HDACPATHWAY | BioCarta | 2 | 25 | 0.002215 | 0.004012 |
| 1 | HSA04630_JAK_STAT_SIGNALING_PATHWAY | KEGG | 3 | 100 | 0.002445 | 0.004346 |
| 12 | METPATHWAY | BioCarta | 3 | 30 | 0.000097 | 0.004639 |
| 13 | METPATHWAY | BioCarta | 3 | 30 | 0.000097 | 0.004639 |
| 14 | METPATHWAY | BioCarta | 3 | 30 | 0.000097 | 0.004639 |
| 1 | AT1RPATHWAY | BioCarta | 2 | 31 | 0.003402 | 0.005832 |
| 1 | HSA04150_MTOR_SIGNALING_PATHWAY | KEGG | 2 | 31 | 0.003402 | 0.005832 |
| 7 | METPATHWAY | BioCarta | 3 | 30 | 0.000132 | 0.006336 |
| 1 | HSA04930_TYPE_II_DIABETES_MELLITUS | KEGG | 2 | 34 | 0.004086 | 0.006881 |
| 1 | HSA04070_PHOSPHATIDYLINOSITOL_SIGNALING_SYSTEM | KEGG | 2 | 38 | 0.005089 | 0.008423 |
| 11 | HSA05214_GLIOMA | KEGG | 3 | 50 | 0.000136 | 0.009527 |
| 11 | HSA04012_ERBB_SIGNALING_PATHWAY | KEGG | 3 | 67 | 0.000327 | 0.011448 |
| 1 | NFATPATHWAY | BioCarta | 2 | 45 | 0.007090 | 0.011536 |
| 1 | PPARAPATHWAY | BioCarta | 2 | 46 | 0.007401 | 0.011842 |
| 1 | HSA04662_B_CELL_RECEPTOR_SIGNALING_PATHWAY | KEGG | 2 | 47 | 0.007718 | 0.012147 |
| 1 | HSA05221_ACUTE_MYELOID_LEUKEMIA | KEGG | 2 | 49 | 0.008371 | 0.012961 |
| 3 | GLEEVECPATHWAY | BioCarta | 2 | 20 | 0.001104 | 0.013025 |
| 3 | IL6PATHWAY | BioCarta | 2 | 20 | 0.001104 | 0.013025 |
| 1 | HSA04370_VEGF_SIGNALING_PATHWAY | KEGG | 2 | 51 | 0.009048 | 0.013787 |
| 3 | TPOPATHWAY | BioCarta | 2 | 23 | 0.001464 | 0.014396 |
| 12 | CREBPATHWAY | BioCarta | 2 | 22 | 0.002132 | 0.016530 |
| 12 | CTLA4PATHWAY | BioCarta | 2 | 15 | 0.000981 | 0.016530 |
| 12 | ECMPATHWAY | BioCarta | 2 | 19 | 0.001587 | 0.016530 |
| 12 | EGFPATHWAY | BioCarta | 2 | 25 | 0.002755 | 0.016530 |
| 12 | ERK5PATHWAY | BioCarta | 2 | 16 | 0.001119 | 0.016530 |
| 12 | ERKPATHWAY | BioCarta | 2 | 25 | 0.002755 | 0.016530 |
| 12 | GHPATHWAY | BioCarta | 2 | 24 | 0.002539 | 0.016530 |
| 12 | GLEEVECPATHWAY | BioCarta | 2 | 20 | 0.001760 | 0.016530 |
| 12 | IGF1PATHWAY | BioCarta | 2 | 18 | 0.001422 | 0.016530 |
| 12 | INSULINPATHWAY | BioCarta | 2 | 19 | 0.001587 | 0.016530 |
| 12 | NGFPATHWAY | BioCarta | 2 | 17 | 0.001266 | 0.016530 |
| 12 | NKCELLSPATHWAY | BioCarta | 2 | 15 | 0.000981 | 0.016530 |
| 12 | PDGFPATHWAY | BioCarta | 2 | 25 | 0.002755 | 0.016530 |
| 12 | TPOPATHWAY | BioCarta | 2 | 23 | 0.002331 | 0.016530 |
| 13 | CREBPATHWAY | BioCarta | 2 | 22 | 0.002132 | 0.016530 |
| 13 | CTLA4PATHWAY | BioCarta | 2 | 15 | 0.000981 | 0.016530 |
| 13 | ECMPATHWAY | BioCarta | 2 | 19 | 0.001587 | 0.016530 |
| 13 | EGFPATHWAY | BioCarta | 2 | 25 | 0.002755 | 0.016530 |
| 13 | ERK5PATHWAY | BioCarta | 2 | 16 | 0.001119 | 0.016530 |
| 13 | ERKPATHWAY | BioCarta | 2 | 25 | 0.002755 | 0.016530 |
| 13 | GHPATHWAY | BioCarta | 2 | 24 | 0.002539 | 0.016530 |
| 13 | GLEEVECPATHWAY | BioCarta | 2 | 20 | 0.001760 | 0.016530 |
| 13 | IGF1PATHWAY | BioCarta | 2 | 18 | 0.001422 | 0.016530 |
| 13 | INSULINPATHWAY | BioCarta | 2 | 19 | 0.001587 | 0.016530 |
| 13 | NGFPATHWAY | BioCarta | 2 | 17 | 0.001266 | 0.016530 |
| 13 | NKCELLSPATHWAY | BioCarta | 2 | 15 | 0.000981 | 0.016530 |
| 13 | PDGFPATHWAY | BioCarta | 2 | 25 | 0.002755 | 0.016530 |
| 13 | TPOPATHWAY | BioCarta | 2 | 23 | 0.002331 | 0.016530 |
| 14 | CREBPATHWAY | BioCarta | 2 | 22 | 0.002132 | 0.016530 |
| 14 | CTLA4PATHWAY | BioCarta | 2 | 15 | 0.000981 | 0.016530 |
| 14 | ECMPATHWAY | BioCarta | 2 | 19 | 0.001587 | 0.016530 |
| 14 | EGFPATHWAY | BioCarta | 2 | 25 | 0.002755 | 0.016530 |
| 14 | ERK5PATHWAY | BioCarta | 2 | 16 | 0.001119 | 0.016530 |
| 14 | ERKPATHWAY | BioCarta | 2 | 25 | 0.002755 | 0.016530 |
| 14 | GHPATHWAY | BioCarta | 2 | 24 | 0.002539 | 0.016530 |
| 14 | GLEEVECPATHWAY | BioCarta | 2 | 20 | 0.001760 | 0.016530 |
| 14 | IGF1PATHWAY | BioCarta | 2 | 18 | 0.001422 | 0.016530 |
| 14 | INSULINPATHWAY | BioCarta | 2 | 19 | 0.001587 | 0.016530 |
| 14 | NGFPATHWAY | BioCarta | 2 | 17 | 0.001266 | 0.016530 |
| 14 | NKCELLSPATHWAY | BioCarta | 2 | 15 | 0.000981 | 0.016530 |
| 14 | PDGFPATHWAY | BioCarta | 2 | 25 | 0.002755 | 0.016530 |
| 14 | TPOPATHWAY | BioCarta | 2 | 23 | 0.002331 | 0.016530 |
| 1 | HSA04540_GAP_JUNCTION | KEGG | 2 | 61 | 0.012790 | 0.019184 |
| 1 | HSA04210_APOPTOSIS | KEGG | 2 | 62 | 0.013196 | 0.019489 |
| 7 | CREBPATHWAY | BioCarta | 2 | 22 | 0.002595 | 0.020103 |
| 7 | CTLA4PATHWAY | BioCarta | 2 | 15 | 0.001196 | 0.020103 |
| 7 | ECMPATHWAY | BioCarta | 2 | 19 | 0.001932 | 0.020103 |
| 7 | EGFPATHWAY | BioCarta | 2 | 25 | 0.003350 | 0.020103 |
| 7 | ERK5PATHWAY | BioCarta | 2 | 16 | 0.001364 | 0.020103 |
| 7 | ERKPATHWAY | BioCarta | 2 | 25 | 0.003350 | 0.020103 |
| 7 | GHPATHWAY | BioCarta | 2 | 24 | 0.003088 | 0.020103 |
| 7 | GLEEVECPATHWAY | BioCarta | 2 | 20 | 0.002143 | 0.020103 |
| 7 | IGF1PATHWAY | BioCarta | 2 | 18 | 0.001732 | 0.020103 |
| 7 | INSULINPATHWAY | BioCarta | 2 | 19 | 0.001932 | 0.020103 |
| 7 | NGFPATHWAY | BioCarta | 2 | 17 | 0.001543 | 0.020103 |
| 7 | NKCELLSPATHWAY | BioCarta | 2 | 15 | 0.001196 | 0.020103 |
| 7 | PDGFPATHWAY | BioCarta | 2 | 25 | 0.003350 | 0.020103 |
| 7 | TPOPATHWAY | BioCarta | 2 | 23 | 0.002837 | 0.020103 |
| 1 | HSA05222_SMALL_CELL_LUNG_CANCER | KEGG | 2 | 67 | 0.015311 | 0.022271 |
| 12 | INTEGRINPATHWAY | BioCarta | 2 | 30 | 0.003960 | 0.022363 |
| 13 | INTEGRINPATHWAY | BioCarta | 2 | 30 | 0.003960 | 0.022363 |
| 14 | INTEGRINPATHWAY | BioCarta | 2 | 30 | 0.003960 | 0.022363 |
| 11 | CREBPATHWAY | BioCarta | 2 | 22 | 0.001008 | 0.023525 |
| 3 | BIOPEPTIDESPATHWAY | BioCarta | 2 | 36 | 0.003584 | 0.026433 |
| 3 | IL2RBPATHWAY | BioCarta | 2 | 34 | 0.003200 | 0.026433 |
| 1 | HSA04620_TOLL_LIKE_RECEPTOR_SIGNALING_PATHWAY | KEGG | 2 | 74 | 0.018506 | 0.026516 |
| 12 | IL2RBPATHWAY | BioCarta | 2 | 34 | 0.005072 | 0.027049 |
| 13 | IL2RBPATHWAY | BioCarta | 2 | 34 | 0.005072 | 0.027049 |
| 14 | IL2RBPATHWAY | BioCarta | 2 | 34 | 0.005072 | 0.027049 |
| 12 | FCER1PATHWAY | BioCarta | 2 | 35 | 0.005370 | 0.027131 |
| 13 | FCER1PATHWAY | BioCarta | 2 | 35 | 0.005370 | 0.027131 |
| 14 | FCER1PATHWAY | BioCarta | 2 | 35 | 0.005370 | 0.027131 |
| 7 | INTEGRINPATHWAY | BioCarta | 2 | 30 | 0.004811 | 0.027167 |
| 1 | HSA04912_GNRH_SIGNALING_PATHWAY | KEGG | 2 | 76 | 0.019467 | 0.027483 |
| 1 | HSA04670_LEUKOCYTE_TRANSENDOTHELIAL_MIGRATION | KEGG | 2 | 83 | 0.022997 | 0.031996 |
| 7 | IL2RBPATHWAY | BioCarta | 2 | 34 | 0.006156 | 0.032831 |
| 7 | FCER1PATHWAY | BioCarta | 2 | 35 | 0.006516 | 0.032924 |
| 12 | TCRPATHWAY | BioCarta | 2 | 40 | 0.006978 | 0.033496 |
| 13 | TCRPATHWAY | BioCarta | 2 | 40 | 0.006978 | 0.033496 |
| 14 | TCRPATHWAY | BioCarta | 2 | 40 | 0.006978 | 0.033496 |
| 12 | HSA04510_FOCAL_ADHESION | KEGG | 3 | 137 | 0.008289 | 0.034599 |
| 12 | HSA05213_ENDOMETRIAL_CANCER | KEGG | 2 | 42 | 0.007676 | 0.034599 |
| 12 | HSA05223_NON_SMALL_CELL_LUNG_CANCER | KEGG | 2 | 43 | 0.008037 | 0.034599 |
| 13 | HSA04510_FOCAL_ADHESION | KEGG | 3 | 137 | 0.008289 | 0.034599 |
| 13 | HSA05213_ENDOMETRIAL_CANCER | KEGG | 2 | 42 | 0.007676 | 0.034599 |
| 13 | HSA05223_NON_SMALL_CELL_LUNG_CANCER | KEGG | 2 | 43 | 0.008037 | 0.034599 |
| 14 | HSA04510_FOCAL_ADHESION | KEGG | 3 | 137 | 0.008289 | 0.034599 |
| 14 | HSA05213_ENDOMETRIAL_CANCER | KEGG | 2 | 42 | 0.007676 | 0.034599 |
| 14 | HSA05223_NON_SMALL_CELL_LUNG_CANCER | KEGG | 2 | 43 | 0.008037 | 0.034599 |
| 2 | GLYCEROPHOSPHOLIPID_METABOLISM | GenMAPP | 1 | 28 | 0.027130 | 0.038758 |
| 2 | HSA00565_ETHER_LIPID_METABOLISM | KEGG | 1 | 21 | 0.020394 | 0.038758 |
| 2 | HSA00600_SPHINGOLIPID_METABOLISM | KEGG | 1 | 20 | 0.019430 | 0.038758 |
| 2 | HSA00980_METABOLISM_OF_XENOBIOTICS_BY_CYTOCHROME_P450 | KEGG | 1 | 26 | 0.025209 | 0.038758 |
| 2 | STATIN_PATHWAY_PHARMGKB | GenMAPP | 1 | 16 | 0.015564 | 0.038758 |
| 7 | TCRPATHWAY | BioCarta | 2 | 40 | 0.008459 | 0.040603 |
| 2 | HSA00380_TRYPTOPHAN_METABOLISM | KEGG | 1 | 34 | 0.032879 | 0.040777 |
| 2 | HSA00564_GLYCEROPHOSPHOLIPID_METABOLISM | KEGG | 1 | 38 | 0.036699 | 0.040777 |
| 4 | BCRPATHWAY | BioCarta | 2 | 32 | 0.001540 | 0.041752 |
| 4 | ERKPATHWAY | BioCarta | 2 | 25 | 0.000937 | 0.041752 |
| 4 | FCER1PATHWAY | BioCarta | 2 | 35 | 0.001842 | 0.041752 |
| 7 | HSA05213_ENDOMETRIAL_CANCER | KEGG | 2 | 42 | 0.009301 | 0.042483 |
| 7 | HSA05223_NON_SMALL_CELL_LUNG_CANCER | KEGG | 2 | 43 | 0.009736 | 0.042483 |
| 10 | HSA00565_ETHER_LIPID_METABOLISM | KEGG | 1 | 21 | 0.027104 | 0.042592 |
| 10 | HSA00600_SPHINGOLIPID_METABOLISM | KEGG | 1 | 20 | 0.025826 | 0.042592 |
| 10 | STATIN_PATHWAY_PHARMGKB | GenMAPP | 1 | 16 | 0.020701 | 0.042592 |
| 8 | HSA00565_ETHER_LIPID_METABOLISM | KEGG | 1 | 21 | 0.027104 | 0.042592 |
| 8 | HSA00600_SPHINGOLIPID_METABOLISM | KEGG | 1 | 20 | 0.025826 | 0.042592 |
| 8 | STATIN_PATHWAY_PHARMGKB | GenMAPP | 1 | 16 | 0.020701 | 0.042592 |
| 12 | HSA05214_GLIOMA | KEGG | 2 | 50 | 0.010770 | 0.043081 |
| 13 | HSA05214_GLIOMA | KEGG | 2 | 50 | 0.010770 | 0.043081 |
| 14 | HSA05214_GLIOMA | KEGG | 2 | 50 | 0.010770 | 0.043081 |
| 10 | GLYCEROPHOSPHOLIPID_METABOLISM | GenMAPP | 1 | 28 | 0.036015 | 0.045568 |
| 10 | TRYPTOPHAN_METABOLISM | GenMAPP | 1 | 29 | 0.037283 | 0.045568 |
| 8 | GLYCEROPHOSPHOLIPID_METABOLISM | GenMAPP | 1 | 28 | 0.036015 | 0.045568 |
| 8 | TRYPTOPHAN_METABOLISM | GenMAPP | 1 | 29 | 0.037283 | 0.045568 |
| 7 | HSA04510_FOCAL_ADHESION | KEGG | 3 | 137 | 0.011030 | 0.046036 |
| 11 | BIOPEPTIDESPATHWAY | BioCarta | 2 | 36 | 0.002708 | 0.047390 |
| 12 | HSA05211_RENAL_CELL_CARCINOMA | KEGG | 2 | 54 | 0.012494 | 0.047977 |
| 13 | HSA05211_RENAL_CELL_CARCINOMA | KEGG | 2 | 54 | 0.012494 | 0.047977 |
| 14 | HSA05211_RENAL_CELL_CARCINOMA | KEGG | 2 | 54 | 0.012494 | 0.047977 |
| 2 | CELL_GROWTH_AND_OR_MAINTENANCE | GO | 1 | 50 | 0.048099 | 0.048099 |
| **Enriched in downregulated paths (BDC2.5/NOD.scid *vs.* NOD.scid)** | | | | | | |
| 9 | PYRIMIDINE_METABOLISM | GenMAPP | 3 | 43 | 0.000010 | 0.000061 |
| 9 | HSA00240_PYRIMIDINE_METABOLISM | KEGG | 3 | 59 | 0.000027 | 0.000080 |
| 3 | HSA00230_PURINE_METABOLISM | KEGG | 3 | 90 | 0.000096 | 0.000334 |
| 3 | PURINE_METABOLISM | GenMAPP | 3 | 74 | 0.000053 | 0.000334 |
| 10 | PYRIMIDINE_METABOLISM | GenMAPP | 3 | 43 | 0.000050 | 0.000448 |
| 10 | HSA00240_PYRIMIDINE_METABOLISM | KEGG | 3 | 59 | 0.000130 | 0.000583 |
| 10 | PURINE_METABOLISM | GenMAPP | 3 | 74 | 0.000256 | 0.000767 |
| 10 | HSA00230_PURINE_METABOLISM | KEGG | 3 | 90 | 0.000458 | 0.001030 |
| 6 | HSA00230_PURINE_METABOLISM | KEGG | 3 | 90 | 0.000458 | 0.001602 |
| 6 | PURINE_METABOLISM | GenMAPP | 3 | 74 | 0.000256 | 0.001602 |
| 3 | PYRIMIDINE_METABOLISM | GenMAPP | 2 | 43 | 0.001130 | 0.002638 |
| 3 | HSA00240_PYRIMIDINE_METABOLISM | KEGG | 2 | 59 | 0.002127 | 0.003722 |
| 1 | PYRIMIDINE_METABOLISM | GenMAPP | 3 | 43 | 0.000203 | 0.005076 |
| 2 | PYRIMIDINE_METABOLISM | GenMAPP | 3 | 43 | 0.000203 | 0.005076 |
| 1 | HSA00240_PYRIMIDINE_METABOLISM | KEGG | 3 | 59 | 0.000522 | 0.006367 |
| 1 | NDKDYNAMINPATHWAY | BioCarta | 2 | 16 | 0.000898 | 0.006367 |
| 1 | PURINE_METABOLISM | GenMAPP | 3 | 74 | 0.001019 | 0.006367 |
| 2 | HSA00240_PYRIMIDINE_METABOLISM | KEGG | 3 | 59 | 0.000522 | 0.006367 |
| 2 | NDKDYNAMINPATHWAY | BioCarta | 2 | 16 | 0.000898 | 0.006367 |
| 2 | PURINE_METABOLISM | GenMAPP | 3 | 74 | 0.001019 | 0.006367 |
| 6 | PYRIMIDINE_METABOLISM | GenMAPP | 2 | 43 | 0.002776 | 0.006477 |
| 9 | PURINE_METABOLISM | GenMAPP | 2 | 74 | 0.003336 | 0.006671 |
| 9 | HSA00230_PURINE_METABOLISM | KEGG | 2 | 90 | 0.004911 | 0.007367 |
| 5 | PYRIMIDINE_METABOLISM | GenMAPP | 3 | 43 | 0.000287 | 0.007469 |
| 8 | PYRIMIDINE_METABOLISM | GenMAPP | 3 | 43 | 0.000287 | 0.007469 |
| 1 | HSA00230_PURINE_METABOLISM | KEGG | 3 | 90 | 0.001803 | 0.009014 |
| 2 | HSA00230_PURINE_METABOLISM | KEGG | 3 | 90 | 0.001803 | 0.009014 |
| 6 | HSA00240_PYRIMIDINE_METABOLISM | KEGG | 2 | 59 | 0.005187 | 0.009077 |
| 5 | HSA00240_PYRIMIDINE_METABOLISM | KEGG | 3 | 59 | 0.000736 | 0.009296 |
| 5 | NDKDYNAMINPATHWAY | BioCarta | 2 | 16 | 0.001119 | 0.009296 |
| 5 | PURINE_METABOLISM | GenMAPP | 3 | 74 | 0.001430 | 0.009296 |
| 8 | HSA00240_PYRIMIDINE_METABOLISM | KEGG | 3 | 59 | 0.000736 | 0.009296 |
| 8 | NDKDYNAMINPATHWAY | BioCarta | 2 | 16 | 0.001119 | 0.009296 |
| 8 | PURINE_METABOLISM | GenMAPP | 3 | 74 | 0.001430 | 0.009296 |
| 5 | HSA00230_PURINE_METABOLISM | KEGG | 3 | 90 | 0.002521 | 0.013109 |
| 8 | HSA00230_PURINE_METABOLISM | KEGG | 3 | 90 | 0.002521 | 0.013109 |
| 9 | NDKDYNAMINPATHWAY | BioCarta | 1 | 16 | 0.020701 | 0.024841 |
| 3 | NDKDYNAMINPATHWAY | BioCarta | 1 | 16 | 0.020701 | 0.028982 |
| 6 | NDKDYNAMINPATHWAY | BioCarta | 1 | 16 | 0.030901 | 0.043261 |
| 3 | HSA05110_CHOLERA_INFECTION | KEGG | 1 | 31 | 0.039815 | 0.046451 |
