# Supplementary material for: Detection of Molecular Paths Associated with Insulitis and Type 1 Diabetes in Non-Obese Diabetic Mouse
Source: PLoS One. 2009 Oct 2;4(10):e7323. doi: 10.1371/journal.pone.0007323 (PMC2749452; doi:10.1371/journal.pone.0007323)
Supplement: Table S3 — Enriched upregulated pathways in insulitis. (0.03 MB DOC) [file pone.0007323.s005.doc]

| **upregulated paths (BDC2.5/NOD vs. NOD)** |  |  |  |  |  |
| --- | --- | --- | --- | --- | --- |
| **Name** | **Size** | **Enrichment Score** | **Nominal**  **p-value** | **FDR**  **q-value** | **Source** |
| BLOOD_CLOTTING_CASCADE | 18 | 0.68 | 0.001816 | 0.1359 | GenMAPP |
