# Supplementary material for: Detection of Molecular Paths Associated with Insulitis and Type 1 Diabetes in Non-Obese Diabetic Mouse
Source: PLoS One. 2009 Oct 2;4(10):e7323. doi: 10.1371/journal.pone.0007323 (PMC2749452; doi:10.1371/journal.pone.0007323)
Supplement: Table S4 — Enriched downregulated pathways in insulitis. (0.09 MB DOC) [file pone.0007323.s006.doc]

| **dowregulated paths (BDC2.5/NOD vs. NOD)** |  |  |  |  |  |
| --- | --- | --- | --- | --- | --- |
| **Name** | **Size** | **Enrichment Score** | **Nominal p-value** | **FDR**  **q-value** | **Source** |
| HSA03010_RIBOSOME | 44 | -0.61 | 0.000466 | 0.0027 | KEGG |
| WNTPATHWAY | 22 | -0.63 | 0.002375 | 0.0252 | BioCarta |
| HSA00071_FATTY_ACID_METABOLISM | 29 | -0.58 | 0.001845 | 0.0291 | KEGG |
| CALCINEURINPATHWAY | 17 | -0.64 | 0.007370 | 0.0392 | BioCarta |
| PROTEASOMEPATHWAY | 21 | -0.61 | 0.004710 | 0.0418 | BioCarta |
| BILE_ACID_BIOSYNTHESIS | 15 | -0.65 | 0.007466 | 0.0425 | GenMAPP |
| PPARAPATHWAY | 46 | -0.49 | 0.002834 | 0.0581 | BioCarta |
| HSA04120_UBIQUITIN_MEDIATED_PROTEOLYSIS | 25 | -0.56 | 0.005639 | 0.0658 | KEGG |
| HSA04110_CELL_CYCLE | 82 | -0.43 | 0.002347 | 0.0765 | KEGG |
| MEF2DPATHWAY | 16 | -0.61 | 0.009468 | 0.0817 | BioCarta |
| HSA04310_WNT_SIGNALING_PATHWAY | 107 | -0.40 | 0.001471 | 0.0951 | KEGG |
| HISTIDINE_METABOLISM | 15 | -0.60 | 0.022326 | 0.0953 | GenMAPP |
| P53HYPOXIAPATHWAY | 17 | -0.59 | 0.020436 | 0.0957 | BioCarta |
| HDACPATHWAY | 25 | -0.54 | 0.012963 | 0.0964 | BioCarta |
| G1_TO_S_CELL_CYCLE_REACTOME | 54 | -0.44 | 0.007260 | 0.0998 | GenMAPP |
| CELL_CYCLE_KEGG | 58 | -0.43 | 0.007106 | 0.1001 | GenMAPP |
| BETA_ALANINE_METABOLISM | 17 | -0.57 | 0.021566 | 0.1048 | GenMAPP |
| VALINE_LEUCINE_AND_ISOLEUCINE_DEGRADATION | 26 | -0.52 | 0.014995 | 0.1048 | GenMAPP |
| VIPPATHWAY | 23 | -0.53 | 0.021790 | 0.1055 | BioCarta |
| HSA04520_ADHERENS_JUNCTION | 61 | -0.43 | 0.003400 | 0.1069 | KEGG |
| GLYCEROLIPID_METABOLISM | 24 | -0.52 | 0.018631 | 0.1163 | GenMAPP |
| CELL_CYCLE | 53 | -0.43 | 0.012470 | 0.1193 | GO |
| MRNA_PROCESSING_REACTOME | 83 | -0.40 | 0.007847 | 0.1195 | GenMAPP |
| CELL_CYCLE_ARREST | 27 | -0.50 | 0.021475 | 0.1234 | GO |
| HSA00190_OXIDATIVE_PHOSPHORYLATION | 86 | -0.39 | 0.008134 | 0.1286 | KEGG |
| NOS1PATHWAY | 19 | -0.52 | 0.039363 | 0.1519 | BioCarta |
| AMIPATHWAY | 15 | -0.55 | 0.051259 | 0.1638 | BioCarta |
| CSKPATHWAY | 15 | -0.55 | 0.051058 | 0.1651 | BioCarta |
| RIBOSOMAL_PROTEINS | 55 | -0.41 | 0.021370 | 0.1656 | GenMAPP |
| HSA00340_HISTIDINE_METABOLISM | 19 | -0.52 | 0.057143 | 0.1719 | KEGG |
| HCMVPATHWAY | 15 | -0.54 | 0.068681 | 0.1826 | BioCarta |
| CIRCADIAN_EXERCISE | 37 | -0.42 | 0.045020 | 0.1898 | GenMAPP |
| HSA00280_VALINE_LEUCINE_AND_ISOLEUCINE_DEGRADATION | 33 | -0.43 | 0.059590 | 0.2124 | KEGG |
| PGC1APATHWAY | 20 | -0.48 | 0.075011 | 0.2152 | BioCarta |
| PROPANOATE_METABOLISM | 23 | -0.47 | 0.072289 | 0.2156 | GenMAPP |
| P53PATHWAY | 16 | -0.52 | 0.084876 | 0.2226 | BioCarta |
| GCRPATHWAY | 16 | -0.51 | 0.092635 | 0.2402 | BioCarta |
| BADPATHWAY | 17 | -0.49 | 0.092421 | 0.2407 | BioCarta |
| BUTANOATE_METABOLISM | 19 | -0.47 | 0.091856 | 0.2499 | GenMAPP |
