# Supplementary material for: Detection of Molecular Paths Associated with Insulitis and Type 1 Diabetes in Non-Obese Diabetic Mouse
Source: PLoS One. 2009 Oct 2;4(10):e7323. doi: 10.1371/journal.pone.0007323 (PMC2749452; doi:10.1371/journal.pone.0007323)
Supplement: Table S5 — Enriched upregulated pathways in type 1 diabetes. (0.05 MB DOC) [file pone.0007323.s007.doc]

| **upregulated paths (BDC2.5/NOD.scid vs. NOD.scid)** |  |  |  |  |  |
| --- | --- | --- | --- | --- | --- |
| **Name** | **Size** | **Enrichment Score** | **Nominal p-value** | **FDR**  **q-value** | **Source** |
| HSA04610_COMPLEMENT_AND_COAGULATION_CASCADES | 52 | 0.62 | 0.000000 | 0.0022 | KEGG |
| HSA04612_ANTIGEN_PROCESSING_AND_PRESENTATION | 33 | 0.66 | 0.000000 | 0.0038 | KEGG |
| HSA04620_TOLL_LIKE_RECEPTOR_SIGNALING_PATHWAY | 74 | 0.54 | 0.000000 | 0.0107 | KEGG |
| HSA04060_CYTOKINE_CYTOKINE_RECEPTOR_INTERACTION | 169 | 0.47 | 0.000000 | 0.0183 | KEGG |
| NKCELLSPATHWAY | 15 | 0.71 | 0.002838 | 0.0310 | BioCarta |
| HSA04940_TYPE_I_DIABETES_MELLITUS | 20 | 0.66 | 0.002753 | 0.0353 | KEGG |
| CTLA4PATHWAY | 15 | 0.67 | 0.009657 | 0.0667 | BioCarta |
| CELL_SURFACE_RECEPTOR_LINKED_SIGNAL_TRANSDUCTION | 100 | 0.43 | 0.001520 | 0.1090 | GO |
| TNFR2PATHWAY | 15 | 0.65 | 0.017364 | 0.1141 | BioCarta |
| HSA04630_JAK_STAT_SIGNALING_PATHWAY | 100 | 0.42 | 0.003979 | 0.1353 | KEGG |
| TH1TH2PATHWAY | 15 | 0.61 | 0.036017 | 0.1593 | BioCarta |
| TOLLPATHWAY | 25 | 0.54 | 0.026235 | 0.1628 | BioCarta |
| 41BBPATHWAY | 15 | 0.60 | 0.042188 | 0.1757 | BioCarta |
| METPATHWAY | 30 | 0.50 | 0.031725 | 0.1946 | BioCarta |
| INTRINSICPATHWAY | 19 | 0.56 | 0.050403 | 0.1960 | BioCarta |
| NFKBPATHWAY | 19 | 0.55 | 0.058741 | 0.2244 | BioCarta |
| BLOOD_CLOTTING_CASCADE | 18 | 0.55 | 0.070517 | 0.2356 | GenMAPP |
