# Supplementary material for: Detection of Molecular Paths Associated with Insulitis and Type 1 Diabetes in Non-Obese Diabetic Mouse
Source: PLoS One. 2009 Oct 2;4(10):e7323. doi: 10.1371/journal.pone.0007323 (PMC2749452; doi:10.1371/journal.pone.0007323)
Supplement: Table S6 — Enriched downregulated pathways in type 1 diabetes. (0.08 MB DOC) [file pone.0007323.s008.doc]

| **downregulated paths (BDC2.5/NOD.scid vs. NOD.scid)** |  |  |  |  |  |
| --- | --- | --- | --- | --- | --- |
| **Name** | **Size** | **Enrichment Score** | **Nominal**  **p-value** | **FDR**  **q-value** | **Source** |
| CELL_CYCLE_KEGG | 58 | -0.57 | 0.000000 | 0.0031 | GenMAPP |
| CELL_CYCLE | 53 | -0.56 | 0.000000 | 0.0073 | GO |
| UBIQUITIN_MEDIATED_PROTEOLYSIS | 20 | -0.67 | 0.000937 | 0.0096 | GenMAPP |
| G1_TO_S_CELL_CYCLE_REACTOME | 54 | -0.53 | 0.000000 | 0.0112 | GenMAPP |
| HSA00190_OXIDATIVE_PHOSPHORYLATION | 86 | -0.49 | 0.000000 | 0.0113 | KEGG |
| P53PATHWAY | 16 | -0.70 | 0.001388 | 0.0144 | BioCarta |
| PROTEASOMEPATHWAY | 21 | -0.64 | 0.000000 | 0.0174 | BioCarta |
| HSA04120_UBIQUITIN_MEDIATED_PROTEOLYSIS | 25 | -0.62 | 0.000473 | 0.0177 | KEGG |
| HSA04110_CELL_CYCLE | 82 | -0.47 | 0.000553 | 0.0211 | KEGG |
| CARM_ERPATHWAY | 19 | -0.63 | 0.004144 | 0.0279 | BioCarta |
| MRNA_PROCESSING_REACTOME | 83 | -0.46 | 0.000000 | 0.0312 | GenMAPP |
| HSA00510_N_GLYCAN_BIOSYNTHESIS | 24 | -0.59 | 0.003738 | 0.0356 | KEGG |
| G2PATHWAY | 18 | -0.62 | 0.004585 | 0.0475 | BioCarta |
| HSA00480_GLUTATHIONE_METABOLISM | 28 | -0.55 | 0.003837 | 0.0527 | KEGG |
| CELL_CYCLE_CHECKPOINT | 23 | -0.58 | 0.005353 | 0.0544 | GO |
| HSA04950_MATURITY_ONSET_DIABETES_OF_THE_YOUNG | 17 | -0.62 | 0.008732 | 0.0547 | KEGG |
| GLUTATHIONE_METABOLISM | 26 | -0.55 | 0.008924 | 0.0631 | GenMAPP |
| HSA00240_PYRIMIDINE_METABOLISM | 59 | -0.46 | 0.002187 | 0.0636 | KEGG |
| CALCINEURINPATHWAY | 17 | -0.61 | 0.011982 | 0.0640 | BioCarta |
| DNA_REPLICATION_REACTOME | 38 | -0.50 | 0.004897 | 0.0658 | GenMAPP |
| N_GLYCAN_BIOSYNTHESIS | 17 | -0.60 | 0.010078 | 0.0672 | GenMAPP |
| HSA01030_GLYCAN_STRUCTURES_BIOSYNTHESIS_1 | 47 | -0.47 | 0.004040 | 0.0696 | KEGG |
| NOS1PATHWAY | 19 | -0.57 | 0.023561 | 0.0880 | BioCarta |
| P53HYPOXIAPATHWAY | 17 | -0.58 | 0.025000 | 0.0968 | BioCarta |
| FLAGELLAR_ASSEMBLY | 15 | -0.57 | 0.043088 | 0.1316 | GenMAPP |
| ATP_SYNTHESIS | 15 | -0.57 | 0.040834 | 0.1323 | GenMAPP |
| TYPE_III_SECRETION_SYSTEM | 15 | -0.57 | 0.043852 | 0.1325 | GenMAPP |
| MEF2DPATHWAY | 16 | -0.56 | 0.039450 | 0.1330 | BioCarta |
| CELLCYCLEPATHWAY | 18 | -0.54 | 0.037140 | 0.1343 | BioCarta |
| ATRBRCAPATHWAY | 15 | -0.56 | 0.051159 | 0.1362 | BioCarta |
| OXIDATIVE_PHOSPHORYLATION | 50 | -0.42 | 0.020176 | 0.1374 | GenMAPP |
| PHOTOSYNTHESIS | 16 | -0.54 | 0.054856 | 0.1475 | GenMAPP |
| PYRIMIDINE_METABOLISM | 43 | -0.42 | 0.025641 | 0.1505 | GenMAPP |
| HSA05110_CHOLERA_INFECTION | 31 | -0.46 | 0.038631 | 0.1512 | KEGG |
| HSA01430_CELL_COMMUNICATION | 60 | -0.40 | 0.021101 | 0.1531 | KEGG |
| HSA03050_PROTEASOME | 22 | -0.47 | 0.075071 | 0.2044 | KEGG |
| HSA04350_TGF_BETA_SIGNALING_PATHWAY | 69 | -0.35 | 0.043780 | 0.2417 | KEGG |
