# Supplementary material for: Detection of Molecular Paths Associated with Insulitis and Type 1 Diabetes in Non-Obese Diabetic Mouse
Source: PLoS One. 2009 Oct 2;4(10):e7323. doi: 10.1371/journal.pone.0007323 (PMC2749452; doi:10.1371/journal.pone.0007323)
Supplement: Table S7 — Excluded cofactors. (0.08 MB DOC) [file pone.0007323.s009.doc]

| **KEGG compound id** | **Primary compound name** | **Secondary compound name** |
| --- | --- | --- |
| C00001 | H2O | water |
| C00003 | NAD+ |  |
| C00004 | NADH |  |
| C00080 | H+ |  |
| C00013 | Pyrophosphate | |
| C00020 | AMP |  |
| C00009 | Orthophosphate | |
| C00008 | ADP |  |
| C00010 | Coenzyme A | |
| C00024 | Acetyl coenzyme A | |
| C00007 | O2 | Oxygen |
| C00006 | NADP+ |  |
| C00005 | NADPH |  |
| C00390 | CoQH2 | Ubiquinol |
| C00399 | CoQ | Ubiquinone |
| C14118 | Naphthyl-2-hydroxymethyl-succinyl CoA | |
| C00007 | Thiamin monophosphate | |
| C00091 | Succinyl coenzyme A | |
| C00026 | 2-Oxoglutarate | |
| C00857 | Deamino-NAD+ | |
| C00027 | H2O2 | Hydrogen peroxide |
| C00030 | AH2 | Reduced acceptor |
| C00019 | Acylcarnitine | S-Adenosyl-L-methionine |
| C00035 | GDP | Guanosine diphosphate |
| C00044 | GTP | Guanosine 5&apos;-triphosphate |
| C00014 | NH3 | Ammonia |
| C00029 | Uridine diphosphate glucose | |
| C00011 | CO2 | Carbon dioxide |
| C00064 | L-Glutamine | |
| C00053 | 3&apos;-Phosphoadenylyl sulfate | |
| C00028 | Acceptor |  |
| C00036 | Oxaloacetate | |
| C00084 | Acetaldehyde | |
| C00016 | FAD | Flavin adenine dinucleotide |
| C00025 | L-Glutamate | |
| C00117 | D-Ribose 5-phosphate | |
| C00103 | D-Glucose 1-phosphate | |
| C00154 | Palmitoyl-CoA | |
| C00033 | Acetic acid | |
| C00055 | Cytidine-5&apos;-monophosphate | |
| C00065 | L-2-Amino-3-hydroxypropionic acid | |
| C00018 | Pyridoxal 5-phosphate | |
| C00051 | Glutathione | |
| C00021 | S-Adenosylhomocysteine | |
| C00582 | Phenylacetyl-CoA | |
| C05598 | Phenylacetylglycine | |
| C05925 | Dihydroneopterin phosphate | |
| C04895 | 2-Amino-4-hydroxy-6-(erythro-1 | 2 |
| C00131 | dATP | Deoxyadenosine triphosphate |
| C00229 | ACP | Acyl-carrier protein |
| C04688 | HMA | (3R)-3-Hydroxytetradecanoyl-[acyl-carrier protein] |
| C14802 | 1-Nitronaphthalene-7 | 8-oxide |
| C00046 | RNAn+1 | Ribonucleic acid |
| C04652 | UDP-2 | 3-bis(3-hydroxytetradecanoyl)glucosamine |
| C00219 | (5Z | 8Z |
| C00105 | UMP | Uridine monophosphate |
|  | | |
| **TransPath comound id** | **Comopund name** |  |
| MO000000328 | ADP |  |
